# Infectious Speciation Revisited: Impact of Symbiont-Depletion on Female Fitness and Mating Behavior of *Drosophila paulistorum*

**DOI:** 10.1371/journal.ppat.1001214

**Published:** 2010-12-02

**Authors:** Wolfgang J. Miller, Lee Ehrman, Daniela Schneider

**Affiliations:** 1 Laboratories of Genome Dynamics, Center of Anatomy and Cell Biology, Medical University of Vienna, Vienna, Austria; 2 Natural Sciences, State University of New York, Purchase College, Purchase, New York, United States of America; Cornell University, United States of America

## Abstract

The neotropical *Drosophila paulistorum* superspecies, consisting of at least six geographically overlapping but reproductively isolated semispecies, has been the object of extensive research since at least 1955, when it was initially trapped mid-evolution in flagrant *statu nascendi*. In this classic system females express strong premating isolation patterns against mates belonging to any other semispecies, and yet uncharacterized microbial reproductive tract symbionts were described triggering hybrid inviability and male sterility. Based on theoretical models and limited experimental data, prime candidates fostering symbiont-driven speciation in arthropods are intracellular bacteria belonging to the genus *Wolbachia*. They are maternally inherited symbionts of many arthropods capable of manipulating host reproductive biology for their own benefits. However, it is an ongoing debate as to whether or not reproductive symbionts are capable of driving host speciation in nature and if so, to what extent. Here we have reevaluated this classic case of infectious speciation by means of present day molecular approaches and artificial symbiont depletion experiments. We have isolated the α-proteobacteria *Wolbachia* as the maternally transmitted core endosymbionts of all *D. paulistorum* semispecies that have coevolved towards obligate mutualism with their respective native hosts. In hybrids, however, these mutualists transform into pathogens by overreplication causing embryonic inviability and male sterility. We show that experimental reduction in native *Wolbachia* titer causes alterations in sex ratio, fecundity, and mate discrimination. Our results indicate that formerly designated *Mycoplasma*-like organisms are most likely *Wolbachia* that have evolved by becoming essential mutualistic symbionts in their respective natural hosts; they have the potential to trigger pre- and postmating isolation. Furthermore, in light of our new findings, we revisit the concept of infectious speciation and discuss potential mechanisms that can restrict or promote symbiont-induced speciation at post- and prezygotic levels in nature and under artificial laboratory conditions.

## Author Summary

The *Drosophila paulistorum* species complex serves as a well-studied model system for evaluating the impact of symbiosis on host speciation since they evolve rapidly and comprise an ancestral, but highly dynamic, reservoir of microbial symbionts. Theory and some experimental evidence suggest that in evolutionary longterm host-symbiont interactions, reproductive parasites might evolve a more benign lifestyle towards mutualism, manipulate sexual mating behavior, and foster host speciation. However, it is an ongoing debate as to whether or not microbial symbionts are capable of driving host speciation in nature and if so, to what extent. Prime candidates are *Wolbachia*, inherited, endosymbiotic bacteria of many arthropods, presently attracting attention as potential biocontrol agents since they affect host reproductive biology. Here we document that all *D. paulistorum* semispecies harbor *Wolbachia* that provide significant fitness benefits to their natural hosts. In semispecies hybrids, however, mutualistic *Wolbachia* turn into pathogens, triggering embryonic lethality and male sterility via overreplication. Besides their impacts on post-mating isolation, we show that in their native *D. paulistorum* hosts *Wolbachia* manipulate sexual behavior by triggering pre-mating isolation via selective mate avoidance, *i.e.* avoiding mates harboring another, incompatible symbiont variant. Our study reveals that endosymbionts can coevolve rapidly with their native hosts and play a significant role in driving natural host speciation.

## Introduction

### Nuclear *vs.* Symbiotic Speciation Mechanisms

In contrast to prezygotic reproductive isolating mechanisms acting before fertilization via mating behavior, postzygotic isolation arises after mating when hybrids are less fit than their parents [1]. In the latter case, the Dobzhansky-Muller model proposes that hybrid incompatibilities, contributing to speciation, are caused by the interaction between nuclear genes that have functionally diverged over time in their respective hybridizing species [2], [3]. Initial direct molecular and genetic evidence for the existence of such Dobzhansky-Muller incompatibility genes recently came from the two sister-species, *D. melanogaster* and *D. simulans* [4]. In this case one of the two incompatibility genes localizes to centromeric heterochromatin - an inherently dynamic part of all eukaryotic genomes known for its accelerated genomic turnover and molecular drive [5], [6]. Genes located within or functionally interacting with hyperdynamic genomic regions, such as nuclear heterochromatin, evolve rapidly and so contribute to hybrid incompatibilities and thereby to speciation [4]–[6]. In this speciation model, however, incompatibility genes are both nuclear genes that have evolved differently after an ancestral populations split.

A second class of rapidly evolving genes possessive of high potential for driving accelerated functional divergence are those genes that are under antagonistic coevolution versus parasites, *i.e*. the reciprocal evolution of host defense and parasite counter-defense mechanisms [7]–[10]. Accordingly, one or more incompatibility genes is encoded by the host nucleus, and the second set by a parasite. In a consistent arms race between invading cytoplasmatically inherited reproductive parasites and a local host population, interacting gene products encoded separately in both organisms evolve rapidly under strong diversifying selection driven by ongoing cyto-nuclear conflicts [11]–[13]. Dense interrelationships between a parasite and its host could functionally weld them together by favoring evolution towards a compensatory partnership where both biological systems finally depend on the presence of the other, and both progress evolutionarily from an antagonistic parasitic to a compensatory mutualist relationship [14], [15]. So it can be expected that repeated, localized episodes of antagonistic and compensatory coevolution may have the potential to diversify and to substructure host populations up to the point where hybrid incompatibilities emerge between them [16], [17]. Increasing empirical evidence and numerous theoretical models predict that transovarially transmitted microbial symbionts that cause cytoplasmic incompatibilities (CI), can have significant impacts as drivers of speciation processes in their natural hosts [12], [16]–[22].

Over the last decades main CI work was focused on the α-proteobacteria *Wolbachia*, a frequent maternally-transmitted endosymbiont of many arthropods and filarian nematodes (reviewed by [23]–[25]). As deduced from classical theoretical models, for example, *Wolbachia* cause unstable equilibrium in the prevalence of the symbiotic infection, rather than stable persistence of infected and uninfected populations [26], [27]. More recent models, however, propose that under specific genetic and environmental conditions *Wolbachia* can promote host speciation in nature [19], [21]. In addition, there are only a few empirical model systems from nature available in literature such as parasitoid wasps of the *Nasonia* sibling species group [28], [29], and mushroom feeding Drosophila species [20], [30]. These data provide clear evidence that *Wolbachia*-induced CI can promote speciation in nature acting in concert with other genetic and/or geographic isolation mechanisms (*see* Discussion).

A recent experimental study, however, shows that artificial loss of *Wolbachia* by antibiotic treatment can have a significant impact on assortative mating behavior of some selected laboratory strains of *D. melanogaster* females, but the mechanism and biological significance in nature remains obscure [31]. Hence the actual role of endosymbionts in speciation in nature must be debated, since we lack appropriate natural and experimentally accessible model systems in early stages of speciation, where conditions that affect reinforcement can be directly tested in laboratories, as well as observed in nature. Finally, with the exception of so-called *mycoplasma-like organisms* (*MLO*s) of *D. paulistorum* [32] and this study, no empirical cases have been reported that endosymbionts are able to cause hybrid male sterility, a hallmark of an early stage in the evolution of postzygotic isolation [1].

Considering the ubiquity of symbiotic interactions and their inherent coevolutionary potential, one could reasonably expect to find experimentally accessible model systems presently under incipient speciation in nature [17]. Based on theoretical considerations [1], [12], [16], [17], [19], [20], [29], [33], [34], requisite for experimentally testing this hypothesis, it is essential to choose a symbiotic model system where both biological components, the host and the symbiont, should fulfill the following main criteria: Firstly, an evolutionary young host species complex in *statu nascendi* ought to be chosen for studying the speciation process in nature. Secondly, this host system should be associated with a fixed endosymbiont with a perfect transmission rate capable of inducing strong bidirectional, postzygotic incompatibilities in hybrids. Finally, this endosymbiont should exert the capacity to trigger prezygotic isolation in order to avoid potential wasteful gene flow between different host infection types. Such candidate model systems are extensively documented in early literature on speciation mechanisms observed in neotropical *Drosophila* hosts [35]–[38].

### The Neotropical *Drosophila paulistorum* Species Complex, a Classic Case of Speciation in *statu nascendi*

**Figure 1:**
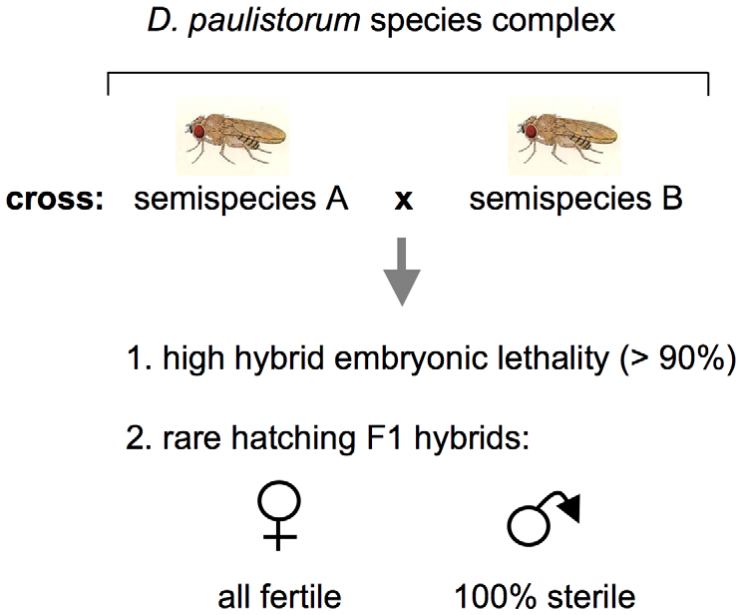
Schematic presentation of incipient speciation among *D. paulistorum* semispecies. Crosses between semispecies give rise in both directions to high hybrid embryonic mortality and complete hybrid male sterility (after [35] as reviewed by [42]).

The first case in literature suggesting that microbial reproductive parasites can play a pivotal role in driving host-speciation dates back to the late 1960s when Ehrman and coworkers discovered endosymbionts that trigger incipient speciation via hybrid inviability and male sterility in a neotropical *Drosophila* group species [39]–[41] reviewed in [42]). The *D. paulistorum* species complex comprises at least six semispecies showing pronounced sexual isolation; matings between these semispecies succeed significantly less frequently than do those within a semispecies [35]–[38]. These are: Amazonian (AM), Andean-Brazilian (AB), Centroamerican (CA), Interior (IN), Orinocan (OR), and Transitional (TR) semispecies. The primary extrinsic mechanism differentiating the *D. paulistorum* semispecies is geographic isolation, albeit incompletely because of consistently overlapping distributions [43]. Three intrinsic isolation mechanisms are operative here - sexual isolation via behavior and hybrid male sterility both in reciprocal crosses [43]. In addition to hybrid male sterility, abnormal pole cell development in early F1 hybrid embryos cause considerable high hybrid inviability [44], [45]; depicted in Figure 1. Since mortality takes place during early stages of embryogenesis [44], [46] and occurs at high frequencies in all hybrids derived from reciprocal matings between the six *D. paulistorum* semispecies [45], [47], this phenotype can be best described as bidirectional cytoplasmic incompatibility, initially described by Laven in the *Culex pipiens* species complex [48].

Transinfection studies have shown that extracts of testes from sterile hybrid males induce a similar sterility syndrome when injected into females of the paternal strain [32]. In 1970 pleomorphic cytoplasmatic symbionts were identified via ultrastructural analyses, and, based on morphological criteria, they were initially designated as *mycoplasma-like microorganisms*, *MLO*s [41]. These intracellular bacteria possess two and sometimes three or four enveloping membranes and were always found in testes, ovaries, and early embryos of both hybrid and nonhybrid *D. paulistorum*, though fewer were counted in nonhybrids. In the male reproductive organs of sterile F1 hybrids however, *MLO*s exist in greater numbers than they do in nonhybrid males [46], [49]. It has been postulated that while each of these semispecies possessed its own *MLO* in a normally benign relationship, in the testes of the hybrid male there is a rapid proliferation of *MLO* and a concomitant breakdown of spermatogenesis [49]. Indeed, hybrid male testes are “sperm-less” microorganismal factories.

Numerous efforts have failed to establish “*MLO*-free” *D. paulistorum* strains using diverse sets of antibiotic treatments [39], [41]. Under mild antibiotic conditions however, Ehrman and colleagues were able to diminish *MLO* titer levels significantly and to partially rescue hybrid male sterility. Based on the observed hypersensitivity of *D. paulistorum* semispecies to different antibiotics, these authors suggested that *MLO*s are maternally transmitted, obligatory microbial symbionts of all *D. paulistorum* semispecies since no truly bacteria-free strain has or had ever been established [39], [41].

Here we report the isolation of close-related α-proteobacteria *Wolbachia*, belonging to the A supergroup, from the six *D. paulistorum* semispecies. Based on their intriguing morphological alikeness with *MLO*s, their tight germline associations, and their striking similarities in infection-titer dynamics in hybrids, *Wolbachia* are most likely identical to formerly designated “*MLO*s” by Ehrman and coworkers [41]. Furthermore repeated attempts via microbial universal 16S rRNA screening and direct sequencing have failed to isolate any other germline-associated microbes besides *Wolbachia*. Hence such “*MLO*s” of previous *D. paulistorum* literature should be reconsidered, from now onward, as members of the invertebrate-colonizing genus *Wolbachia*. We have furthermore reevaluated symbiotic interactions between *Wolbachia* and their natural *D. paulistorum* semispecies hosts by studying phenotypic traits appearing upon partial symbiont-depletion via antibiotics, traits such as female fecundity, sex ratios, and most significantly, female mating behavior.

## Results

### Isolation and Characterization of *Wolbachia* as the Unique Endosymbiont Associated with the *D. paulistorum* Germline

Since “*MLO*s” have been diagnosed as maternally-transmitted endosymbionts that accumulate in testes, ovaries and early embryos [42], [43], [46], [49] we have surveyed the germline-associated microbial diversity of the *D. paulistorum* OR semispecies by applying universal 16S rRNA PCR [50] on DNA of fertilized early embryos derived from sterilized eggs. This approach avoids contaminations with gut bacteria, focusing our survey on vertically-transmitted endosymbionts. In total, twenty amplified 16S rRNA consensus fragments of 437 bp derived from five independent PCR reactions were cloned and sequenced. All reads from early embryos of the OR semispecies were identical to the *Wolbachia* 16S rRNA gene of the sibling species *D. willistoni* (GenBank accession number DQ412086).

**Figure 2:**
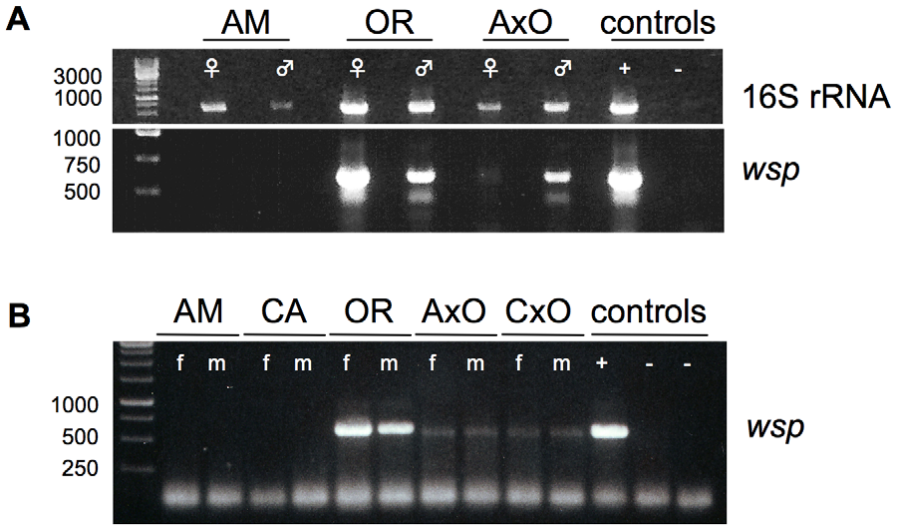
Presence of germline-associated microbes in *D. paulistorum* semispecies and their sterile hybrids. (**A**) PCR on total DNA extracted from each 15 pairs of ovaries (♀) and testes (♂) from ten day-old Amazonian (AM), Orinocan (OR) semispecies, and from hybrids (AxO) derived from matings between AM females and OR males. Controls are testes of *D. simulans* infected with *w*Ri *Wolbachia* (+) and *D. melanogaster* strain *w* ^1118^ uninfected (−). PCRs were performed with universal 16S rRNA (upper gel) and *Wolbachia*-specific *wsp* (lower gel) primer. (**B**) *Wolbachia*-specific *wsp* PCR on total DNA from ten flies of *D. paulistorum* semispecies and hybrids of Amazonian (AM), Centroamerican (CA), Orinocan (OR) semispecies, and F1 hybrids derived from crossings of AM females to OR males (AxO) and CA females to OR males (CxO). PCR controls are adults of *D. simulans* infected with *w*Ri *Wolbachia* (+) and two uninfected strains (−), *i.e.*, *D. simulans* STC and *D. melanogaster* strain *w* ^1118^. DNA was extracted from 10-day-old females (f) and males (m).

Furthermore “*MLO*s” have been described earlier as consistently associated symbionts within adult reproductive tissues of all *D. paulistorum* semispecies, specifically over-replicating in testes of reciprocal hybrids [46], [49]. Hence we have dissected under sterile conditions ovaries and testes from mature AM and OR semispecies, plus AxO hybrid flies for DNA extraction. Gonad-specific PCRs were performed with two sets of universal 16S rRNA primer [50], [51] (for details *see* Materials and Methods) on parental and hybrid samples. As shown in Figure 2A (upper gel), gonads of both OR sexes harbor more microbial symbionts than AM semispecies, with weakest signal intensity in AM testes. In AxO hybrid testes, however, 16S rRNA signal intensities were significantly higher than in AM paternal ones (Figure 2A). Consensus 16S PCR fragments of 25 independent reactions derived from reproductive organs of native and hybrid samples (6, 14 and 5 reads for AM, OR and hybrids, respectively) were direct-sequenced. In the six reads derived from AM ovaries and testes double peaks were detected from which one sequence type was found with 100% homology to the 16S rRNA of *w*Wil of *D. willistoni* (position 291–727 and 392–861 in DQ412086, for primer set of [50], [51], respectively). The second type of 16S rRNA, however, uncovered the presence of a second bacteria, belonging to *Acetobacter*. On the other hand all reads of OR (14) and AxO hybrids (5) were identical to the 16S rRNA of *D. willistoni* (GenBank accession number HQ185362 and HQ185363, respectively) and no other bacteria could be recovered. The identification of *Acetobacter* in dissected gonads from AM samples demonstrates the sensitivity of our detection system since this semispecies habors *Wolbachia* at extreme low density compared to OR and AxO hybrids (see below). In the latter two samples we never amplified *Acetobacter* from dissected gonads. Furthermore *Acetobacter* are free-living bacteria generally found as part of the gut flora in insects that have never been associated with any reproductive phenotype in literature. We therefore assume that the *Acetobacter* detected in AM samples can be regarded most likely as gut contaminant from gonad dissections. These data strongly implicate that *Wolbachia* are the unique germline associated symbiont in *D. paulistorum* semispecies.

In order to correlate quantitative shifts of universal microbial rRNA titer levels in hybrid testes with our candidate *Wolbachia*, PCRs were performed on same DNA samples with a diagnostic *wsp* primer set. This approach is known as a robust and reliable *Wolbachia*-specific diagnostic marker targeting selectively the gene *wsp-A* encoding the Wolbachia surface protein-A [52]. Similar to observed 16S PCR patterns mentioned above, OR and AM semispecies differ dramatically in their gonad-associated symbiont titer levels (Figure 2A, lower gel). Whereas gonads of both OR sexes are highly infected with *Wolbachia*, via standard PCR methods no clear signals were obtained from ovaries and testes of AM semispecies. In contrast, ovaries of AxO hybrids exhibit weak but clear *Wolbachia* signals of expected size and much stronger ones in AxO testes. Both gonad-specific PCR assays were performed in independent triplicates exhibiting similar patterns of diagnostic *Wolbachia* intensities as mentioned above (data not shown). We therefore conclude that *Wolbachia* are most likely the exclusive germline associated symbiont that overreplicates in hybrid testes of *D. paulistorum* semispecies.

Next we have monitored the presence of *Wolbachia* in individual adult females and males from AM, CA, and OR semispecies by *wsp* PCR. Furthermore, mature adult hybrids (AxO and CxO) obtained by mating AM and CA females to OR males respectively, were also assayed (Figure 2B). Adults of *D. paulistorum* semispecies, however, differ significantly in their respective *Wolbachia* titer levels. Under these assay conditions, no signals could be detected in adult males and females of AM and CA semispecies, whereas OR flies exert bright *wsp* PCR signals similar to the intensity of *w*Ri of *D. simulans* (Figure 2B). Hybridizing PCR blots with a *wsp*-specific internal probe [53], however, definitely verified the presence of *Wolbachia* in adults of all six semispecies, *i.e*., AB, AM, CA, IN, OR and TR, although five of them harbor extremely low-titer infections, beyond the limit of standard PCR detection methods (Figure S1).

In addition we have performed PCR screens with a second *Wolbachia*-specific PCR marker set, the multi-copy transposon *IS*5 [54], [55], on at least fifty single fly reactions per semispecies. DNA extractions and PCR reactions were set up under sterile conditions in independent replicates of ten males and ten females per semispecies, plus two simultaneously extracted single flies of the uninfected line *D. simulans* STC. Only such *IS*5 PCR data sets were counted where both negative samples were found uninfected. In total, all three hundred individuals of the six *D. paulistorum* semispecies were diagnosed as *Wolbachia* infected (data not shown). Hence the high infection status suggests perfect maternal transmission of mainly low-titer *Wolbachia* in *D. paulistorum* superspecies.

As reported earlier, hybrid male sterility is caused by overreplication of maternally transmitted “*MLO*s” in the testes of interstrain hybrids that provoke breakdown of spermatogenesis and finally generate completely immotile gametes [41]. Therefore, loss of bacterial replication control in F1 testes should be correlated with a significant increase in *Wolbachia* infection titers in germlines of hybrids. Consequently, we have performed *Wolbachia-*specific *wsp* PCR on DNA from adult hybrid F1 females and males derived from crosses between low-titer AM and CA mothers and high-titer OR fathers. As shown in Figure 2B, *Wolbachia* levels shift quantitatively from extremely low-titer in AM and CA mothers, towards intermediate-titer infections in F1 hybrid of both sexes (AxO and CxO).

**Figure 3:**
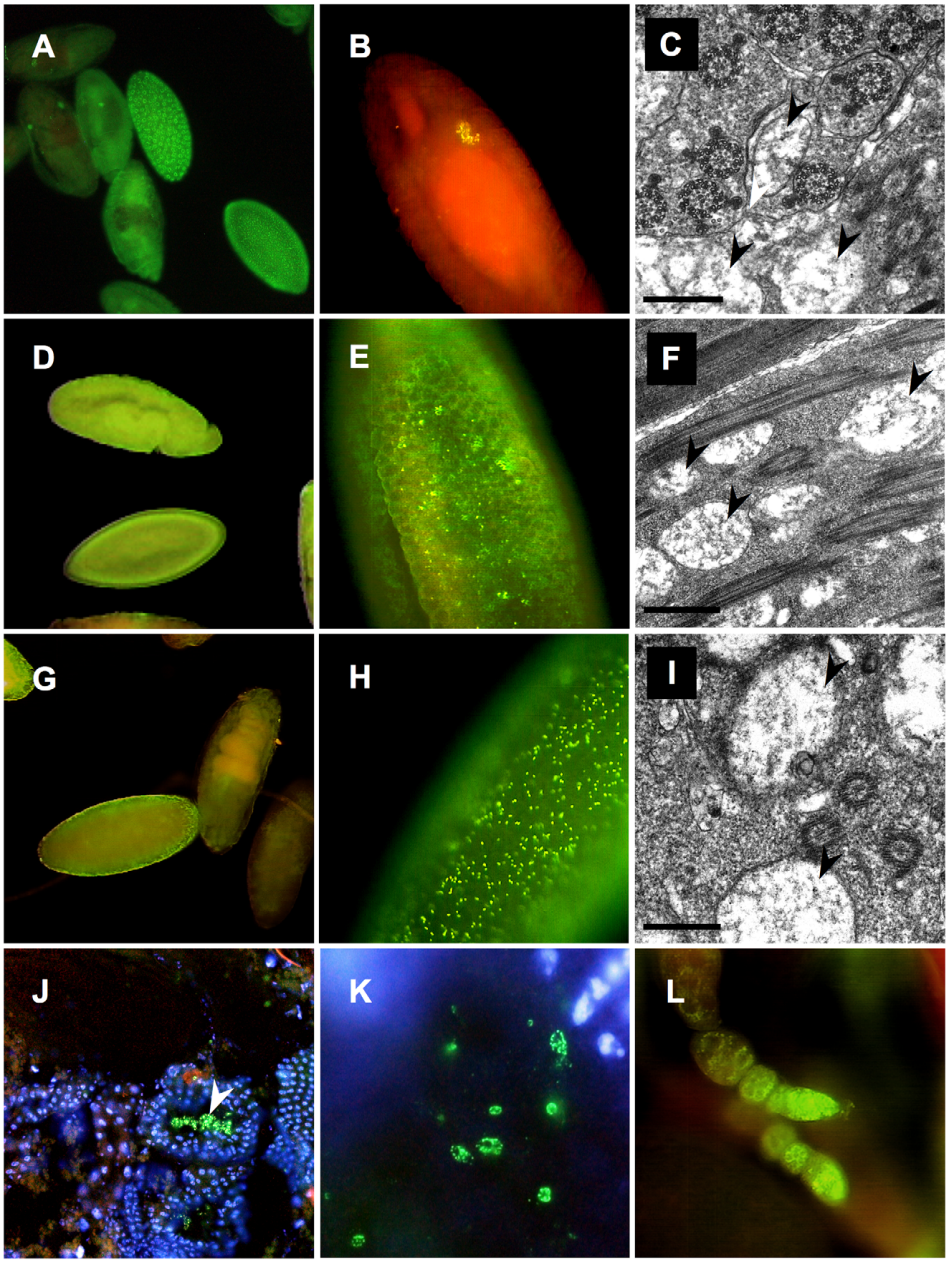
Distribution of *Wolbachia* during development of *D. paulistorum* semispecies. OR (**A–C**), AM (**D–F,L**) and AxO hybrids (**G–K**) of AM females and OR males. Whole-mount immunostainings with the *Wolbachia*-specific anti-WSP antibody (yellow/green) were performed on embryos (**A,B,D,E**, and **G,H**), testes (**J,K**), and ovaries (**L**); DNA is counter-stained with DAPI (blue), or propidium iodide (red) following the protocol of [59]. Transmission electron microscopy of rod-shaped pleomorphic *Wolbachia* cells (arrows) in the testes of fertile OR (**C**) and AM semispecies (**F**), plus sterile AxO hybrid males (**I**). Symbiont morphology and density in reproductive host tissues corroborate earlier pictures published in [41], [49]. In OR semispecies high-titer *Wolbachia* accumulate in early blastodermal stages (**A**); in further differentiating embryos symbionts selectively target the primordial germ cells of OR (**B**). Presence of *Wolbachia* in OR testes associated with developing spermatids (**C**). In AM semispecies *Wolbachia* are present at very low-titer levels presumably in somatic and germline cells during blastodermal, gastrulating and late embryonic development (**D**). Higher magnification of bastodermal AM embryos clearly shows low density of the symbiont (**E**). Signal intensities are clearly enhanced in F1s of AxO derived from matings between AM females and OR males, suggesting overreplication of the maternally-trasmitted symbiont in hybrids (**G,H**). *Wolbachia* immunostainings on cryosections of testes of AxO hybrids in transversal sections (**J**), and during spermatid development (**K**). Presence of *Wolbachia* during *D. paulistorum* oogenesis (**L**).

Furthermore ultrastructural analyses and whole mount immunostainings were performed applying the *Wolbachia*-specific WSP antibody on fixed embryos, testes and ovaries of native AM and OR hosts and AxO hybrids (Figure 3) Similar to data obtained from 16S rRNA and *wsp* PCR assays, OR and AM semispecies differ significantly in their respective symbiont titer levels during development. Compared to OR semispecies that exert strong *Wolbachia* signals in early blastodermal stages (Figure 3A), but further on concentrate specifically in primordial germline cells, PGC (Figure 3B), AM semispecies show only faint but universal staining signals during embryogenesis (Figure 3D,3E). Presence of *Wolbachia* was also undoubtedly detected in AM ovaries (Figure 3L). In contrast, in AxO hybrids, however, where the symbiont was inherited from low-titer AM mothers (Figure 3E), *Wolbachia* densities were significantly higher in blastodermal stages (Figure 3G,3H), and clear signals were detectable in hybrid testes within spermatids (Figure 3J,3K). Furthermore ultrastructural analyses on testes of OR (Figure 3C) AM (Figure 3F) and AxO hybrids (Figure 3I) confirmed the general presence of endosymbionts during spermatogenesis with multiple membrane layers morphologically resembling *Wolbachia* and earlier descriptions [41], [49]. Hence we conclude, that intracellular *Wolbachia* are identical to earlier designated “*MLO*s”, the germline-associated core endosymbionts of *D. paulistorum* semispecies, and therefore pose most likely the unique microbial candidate as the causative agent driving incipient speciation in this Drosophila host system.

For *Wolbachia* strain typing, *wsp* sequence data were obtained by PCRs from multiple independent samples per semispecies (embryonic DNA, adults and hybrids) of AM, CA, OR and TR semispecies. Within a semispecies group no sequence polymorphism could be detected. The *wsp* locus of *Wolbachia* is encoding a rapidly evolving outer membrane protein that is under strong diversifying selection and hence permits distinguishing between even closely related *Wolbachia* strains [11], [56], [57], [58]. Based on *wsp* data, CA, TR and OR *Wolbachia* (GenBank accession numbers GQ924888 to GQ924890) are almost identical to the *w*Au strain of *D. simulans* and to ancestral infections found in closely related neotropical *Drosophila* hosts, *D. willistoni*, *D. prosaltans*, and *D. septentriosaltans* [59]. As shown in Table S1 *Wolbachia* of CA and TR differ from OR at one unique diagnostic site in the hypervariable region HVR1 of the WSP protein [58]. Although *wsp* of OR semispecies is identical to the orthologous locus *w*Spt BCI1 of *D. septentriosaltans* and *w*Cer2 of *Rhagoletis cerasi*, the OR infection harbors a unique variant of the hypervariable *VNTR-*105 [57] locus (GenBank accession number GQ924884; and unpublished data).

In AM semispecies however, a different *Wolbachia wsp* sequence identical to the one of *w*Ri of *D. simulans* was found (GenBank accession number GQ924887). Canonical *w*Ri *Wolbachia* were originally isolated from natural populations of *D. simulans* able to induce strong CI when infected males are mated to uninfected females [60]. The close phylogenetic relationship between *w*Ri-like *Wolbachia* of AM *D. paulistorum* and *w*Ri of *D. simulans* was corroborated by sequence data obtained from the non-coding *VNTR-*105 locus [57] with diagnostic base signatures characteristic of the *w*Ri infection (GenBank accession number GQ924885). The phylogenetic relationships between Drosophila hosts and their respective *Wolbachia* symbionts are shown in Figure S2.

### Effect of Mild Antibiotic Treatments on *D. paulistorum* Female Fecundity

Currently, applying Tetracycline at 0.03% final concentration in regular food over up to three generations is the standard procedure for successfully curing a diverse range of *Drosophila* species of their natural symbionts without inducing significant negative fitness effects in their respective hosts [59], [61], [62]; but also *see* [63]). In contrast, *D. paulistorum* semispecies are hypersensitive to standard antibiotic treatments since we have never obtained a single F1 progeny from curing assays of adults at 0.03% Tetracycline (unpublished data). Such results are in agreement with earlier observations that “*MLO*s” are obligatory microbial symbionts of all *D. paulistorum* semispecies and no true bacteria-free strain has ever been established [39], [41], [46], [49], [64], [65].

On the other hand, mild antibiotic assay conditions, 0.01% Tetracycline or 0.01 to 0.2% Rifampicin, allowed us to establish lines of AM, CA, and OR semispecies for more than three generations on antibiotics. Furthermore we have managed to generate hybrid lines between different *D. paulistorum* semispecies that are stable on consistent antibiotic media (0.2% Rifampicin) for more than 12 consecutive generations under sibling mating conditions. These hybrid lines were originated from crosses between antibiotic-treated virgin AM and OR parents producing F1 hybrids of partially fertile males and fertile females. F1 progeny were allowed to mate randomly and have reached F12 at the time this manuscript was written. In contrast, control matings between untreated AM and OR semispecies have never resulted in any F2 hybrids. Detailed description of these “stabilized” hybrid lines will be presented elsewhere in a separate publication as soon as more generations are assayed.

For evaluating the potential impact of artificial symbiont-depletion on rescuing hybrid mortality, we have crossed OR females and AM males. This combination is known to induce complete CI since we have never obtained a single F1 hybrid in repeated attempts in both of our laboratories. Antibiotic treatment of both parents, however, was sufficient to overcome complete CI and to partially rescue embryonic mortality. In these experiments interstrain matings were set up in three replicates of twenty virgin OR females and AM males each, allowed to lay hybrid eggs for 48 hrs. Parents were then discarded and hatching F1 adults were counted after two weeks. Whereas no single F1 fly emerged from control matings (both untreated) between 32 and 39 adult hybrids of both sexes emerged per assay from parents that were derived from artificially symbiont-depleted lines.

Hence similar to earlier reports [39], [41], [49], [65] longterm treatments at 0.01% Tetracycline and up to 0.2% Rifampicin in our laboratory are sufficient to accomplish partial rescue of hybrid male sterility and also to increase hatching rates among these *D. paulistorum* semispecies.

**Figure 4:**
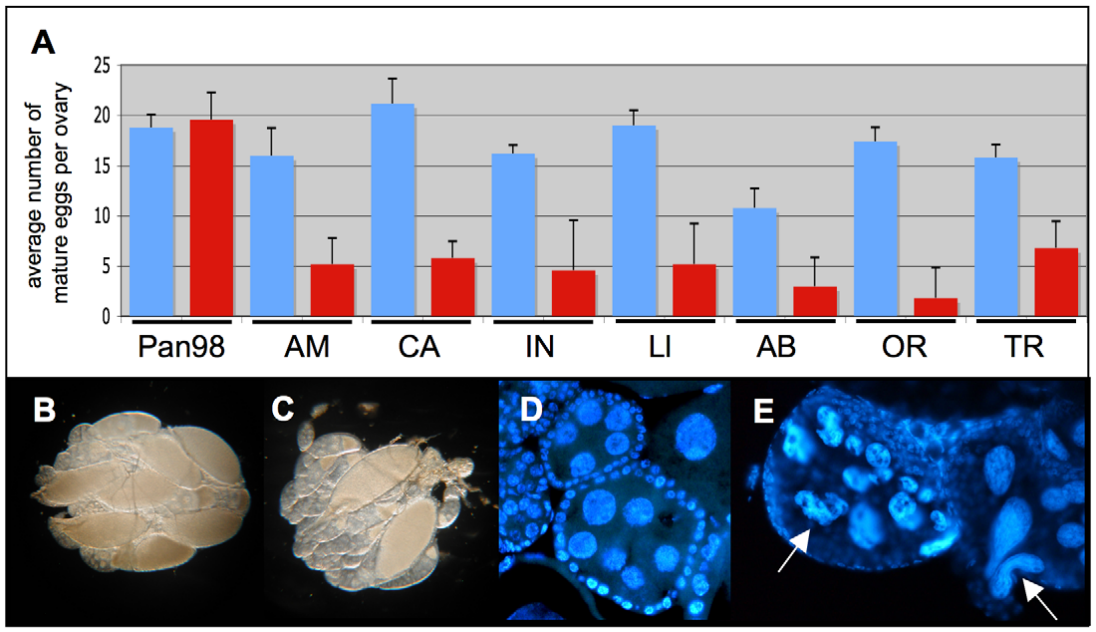
Effect of mild antibiotic treatment on female *Drosophila* fecundity. (**A**) Mean numbers and standard deviations (SD) of mature eggs per pair of ovaries of eight-day-old females (blue bars) and after 15 generations of mild Tetracycline of 0.01% (red bars). Total n = 80. Seven *D. paulistorum* strains were assayed belonging to the six described semispecies Amazonian (AM), Centroamerican (CA), two Interior (IN and Ll), Andean-Brazilian (AB), Orinocan (OR), and Transitional (TR). *D. willistoni* (Pan98) is a control strain that was collected in 1998 in Panama naturally infected with *w*Wil *Wolbachia* [59]. (**B,C**) Effect of mild antibiotic treatment on *D. paulistorum* oogenesis in 10-day-old females in (**B**) untreated OR control and (**C**) after three generations on 0.1% Rifampicin. (**D,E**) DAPI staining of egg chambers of (**D**) untreated and (**E**) treated OR females showing highly abnormally shaped nurse cells (white arrows).

Having determined sublethal dosages of these two antibiotics, Tetracycline and Rifampicin, we have observed that treated *D. paulistorum* females suffer low fecundity. In order to rule out unspecific side effects of antibiotics on fly oogenesis, we have applied the same treatment conditions to their closely related sister species *D. willistoni*, naturally infected with facultative *Wolbachia* [59]. Treated females of all *D. paulistorum* semispecies showed a significant reduction in mature egg numbers in their ovaries, whereas similar longterm treatments at 0.01% Tetracycline of closely related *D. willistoni* controls (Pan98) had no effect on oogenesis (Figure 4A). Scoring of mature eggs followed [66]. In *D. willistoni* average numbers of mature eggs per ovary before and after treatment were 18.8±1.30 and 19.6±2.69, respectively, but in the pool of the seven *D. paulistorum* strains tested numbers dropped down dramatically from averaged 16.63±4.61 in untreated, to 4.61±2.14 in treated ones. Furthermore, Rifampicin treatment at 0.1% final concentration over three generations also resulted in a severe decrease of mature egg numbers in *D. paulistorum* where the vast majority of egg chambers were underdeveloped (Figure 4B, C). Closer inspections, using DAPI staining, showed abnormally shaped, and highly degenerate nurse cell nuclei within treated egg chambers (Figure 4D,4E), suggesting that the low fecundity observed in treated *D. paulistorum* females has been already provoked by defects in cell cycle and/or apoptosis during very early stages of oogenesis. Similar defects in oogenesis upon artificial symbiont depletion have been earlier reported in the parasitic wasp *Asobara tabida* [15]; and *see* Discussion).

### Emergence of Sex Ratio Distortions in Favor of Males upon Partial Symbiont Depletion

In the course of our *D. paulistorum* semispecies depletion assays we have observed that mild antibiotic treatment conditions at 0.01% Tetracycline and 0.01% Rifampicin are capable of skewing sex ratios towards males in AM but not in OR semispecies. In accordance with earlier reports [42], untreated AM and OR semispecies have the usual 1∶1 sex ratio (Table 1). Under mild Tetracycline treatment (0.01% over five generations) however, the AM semispecies produces a highly significant sex bias towards males (ratio males to females  = 1.79, χ^2^ = 43.27; d.f. = 1; *P*<0.0001), whereas the OR semispecies did not deviate from the usual 1∶1 sex ratio (Table 1). Significant excess of males was also observed in AM flies kept for five generations on 0.01% Rifampicin (ratio males to females  = 1.46, χ^2^ = 26.60; d.f. = 1; *P*<0.0001). Like mild Tetracycline treatments, there was no effect of Rifampicin on sex ratios in OR semispecies (ratio males to females  = 1.01, χ^2^ = 0.012; d.f. = 1; *P* = 0.91). However, higher dosages of Rifampicin (0.03% to 0.1%) were sufficient for inducing statistically significant male sex biases (*P*<0.0001) also in OR semispecies (Table 1).

**Table 1:** Effect of Mild Antibiotic Treatments on *D. paulistorum* Sex Ratios.

Amazonian	Males	Females	sex ratio§	χ^2^	*P*
untreated	498	470	1.06	0.81	0.36814
Tet 0.01%	347	194	1.79	43.27	<0.0001
Rif 0.01%	456	313	1.46	26.60	<0.0001
Rif 0.03%	597	430	1.39	27.16	<0.0001
Rif 0.1%	544	372	1.46	32.30	<0.0001

Orinocan	Males	Females	sex ratio	χ^2^	*P*
untreated	520	506	1.03	0.191	0.6621
Tet 0.01%	481	477	1.01	0.017	0.8972
Rif 0.01%	364	361	1.01	0.012	0.9113
Rif 0.03%	518	360	1.44	28.43	<0.0001
Rif 0.1%	526	359	1.47	31.51	<0.0001

**§:** Sex ratio in the *D. paulistorum* semispecies Amazonian (AM) and Orinocan (OR) emerging from regular food (untreated) and two independent antibiotic-treatments with Tetracycline (Tet) and Rifampicin (Rif). Total number of flies counted for two weeks from initial day of hatching was 8,693 divided into 4,851 males and 3,842 females. Untreated AM and OR semispecies have the usual 1∶1 sex ratio (also *see* [42] and references therein). Both antibiotic treatments were performed either over five generations on 0.01% Tet or 0.01% Rif; or over three generations on 0.03% and 0.1% Rif.

Hence, the two *D. paulistorum* semispecies tested on antibiotics are both highly permissive in shifting sex ratios towards males, but in drug dosage-dependent manners. Whereas sex ratio distortion becomes manifested in AM semispecies already at low concentrations of antibiotics, OR semispecies require more severe treatment conditions for inducing a similar phenotype. Dosage-dependent induction of this sex ratio phenotype is directly correlated with our finding that OR semispecies harbor high-titer *Wolbachia*, while AM semispecies are only infected at low densities. We thus assume that in OR semispecies, higher dosages of antibiotics are necessary to overcome a specific threshold level, critical to affecting symbiont-density significantly, and capable for inducing sex ratio distortions in favor of males. Excess of adult males in treated *D. paulistorum* lines can potentially be explained by higher sensitivities of females than males to partial symbiont depletion, resulting in enhanced embryonic or larval mortality of females. Similar male-biased distortion phenotypes upon artificial microbial depletion have been reported earlier in host - symbiont systems where obligate, maternally-transmitted endosymbionts serve obligate mutualistic functions in filarian nematodes [67], [68]; and *see* Discussion. Hence, this report is, to our knowledge, the first case in literature demonstrating the manifestation of a male-biased sex ratio distortion phenotype upon artificial symbiont depletion in arthropods.

Since all tested strains covering the six *D. paulistorum* semispecies are old laboratory lines, collected before the 1960s, it is possible that in the course of their long term rearing conditions these lines might have picked up *Wolbachia* artificially and/or that relaxed selection in the laboratory has led to artificial co-adaptations between host and symbiont. In order to overcome this criticism we have included two more recent *D. paulistorum* lines that were collected in Southern Brazil in 2003, (*see* Materials and Methods). These two lines, POA1 and POA10 were established from isofemales collected in Porto Alegre, Rio Grande. Both lines belong to the Andean-Brazilian (AB) semispecies group, since they give rise to fertile male hybrids when mated reciprocally to the Mesitas strain (AB); unpublished data. Based on *wsp* marker sequences both POA strains are infected with the same *Wolbachia* type (data not shown), but differ significantly in their respective titer levels (Figure S3). Whereas POA1 adults harbor high-titer infections similar to Orinocan semispecies, *Wolbachia* levels are very low in POA10 adults, similar to most other *D. paulistorum* semispecies. We therefore conclude that both low- and high-titer *Wolbachia* are natural symbionts of *D. paulistorum* semispecies. More data from present-day samples from wild populations will be important to deepen our understanding in the temporal and spatial host-symbiont dynamics and their present impact on incipient speciation in nature.

### Effects of *Wolbachia*-Depletion on Premating Isolation and Female Mate Avoidance

Reinforcement is the process by which postmating isolation acts as a direct selective pressure fostering the evolution of premating isolation in areas of sympatry [2], [69]. In this respect, reinforcement is judged the final most efficient stage in speciation processes that block gene flow to incipient species via mating discrimination. Understanding how sexual isolation evolves requires that we capture the process before it has reached completion. Therefore, the *D. paulistorum* species complex, a cluster of semispecies in *statu nascendi*, serves as a perfect model system for elucidating the mechanisms and dynamics of incipient sexual isolation [42].

Based on our data, we propose that different semispecies of *D. paulistorum* harbor mutually incompatible fixed *Wolbachia* strains that cause postzygotic isolation by strong bidirectional CI and hybrid male sterility in the laboratory [42]. In reciprocal crosses between any of the six semispecies, F1 hybrid females are fertile and - under laboratory conditions - gene flow is possible between semispecies. In nature, however, incompatible matings between semispecies are avoided by female mating choice and courtship behavior [65], [70]. In the *D. paulistorum* species-complex sexual isolation between sympatric semispecific strains is significantly greater than sexual isolation between allopatric semispecific strains [37], [71].

**Figure 5:**
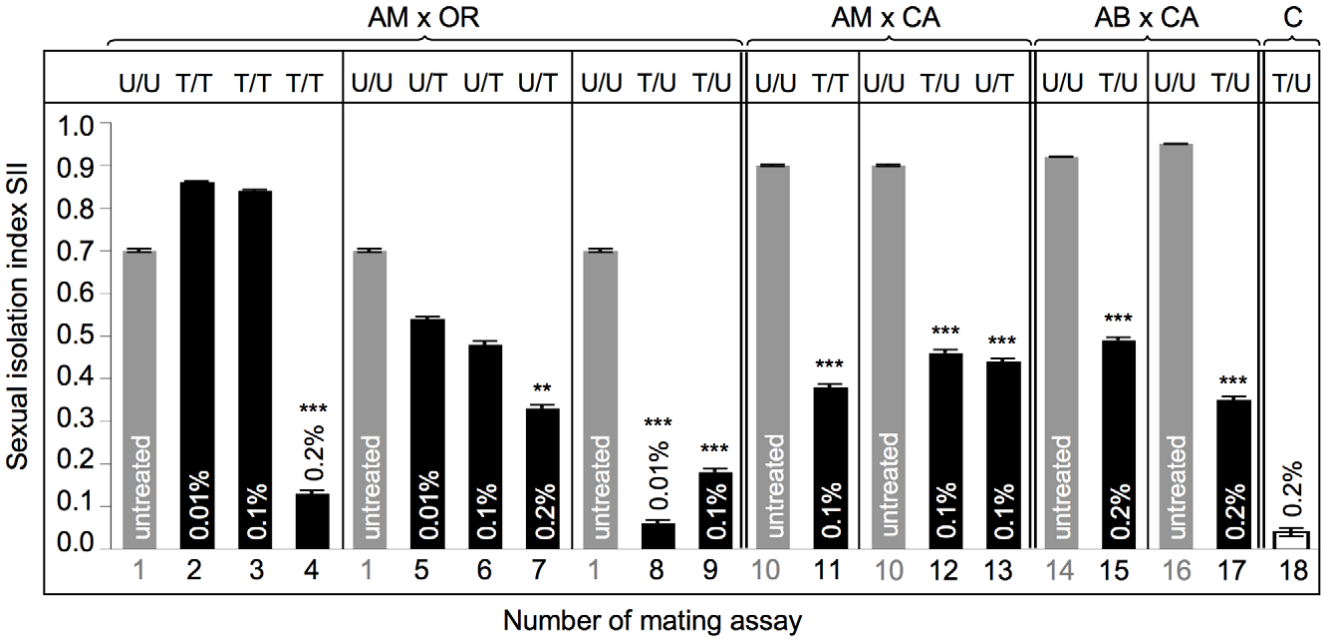
Mating preferences in combinations between untreated and treated heterogamic pairs of *D. paulistorum* semispecies. y-axis represents sexual isolation index (SII); number of mating assay (1–18) is shown on x-axis (corresponding to assay numbers in Table S2). Grey bars indicate untreated controls; black bars indicate assays with Rifampicin treated flies: Tested lines were kept on 0.01% Rifampicin for ten generations; or on 0.1% and 0.2% Rifampicin for five generations. SII and standard error (SE) were determined following [75]. For each array five replicates and 120 matings were scored (12A ♀♀ +12B ♀♀ +12A ♂♂ +12B ♂♂ differentiated by rotated wing clips) for each row, totaling 2,160 matings. Two-tailed *P* values were calculated by comparing SIIs of untreated and treated pairs of mating choice assays by Fisher's exact test. Statistically significant results are indicated by one, two or three asterisks, *i.e*., *P*<0.05; *P*<0.01 and *P*<0.001, respectively. Abbreviations: Amazonian (AM); Centroamerican (CA); Orinocan (OR) and Andean-Brazilian (AB) semispecies (lines POA1 and POA10). U =  untreated; T =  treated with antibiotics.

In order to evaluate the potential role of *Wolbachia* mutualists on sexual isolation we have performed mating choice assays between naturally infected and artificially depleted *D. paulistorum* strains of AB, AM, CA and OR semispecies. Sexual isolation indices (SIIs) were measured between combinations of heterogamic pairs of AM, CA, OR and AB (line POA1 and POA10) semispecies [71], [72], before and after Rifampicin treatments for at least five consecutive generations. For AM x OR interstrain assays, lines derived from three different concentrations of the antibiotic, *i.e*., low-Rif at 0.01% and high-Rif at 0.1% and 0.2%, whereas AM x CA and AB x CA assays were performed at high-Rif only. As shown in Figure 5, control assays between untreated (U), naturally infected, pairs of AM and OR semispecies document high sexual isolation (assay 1, SII_AM_^U^_X OR_^U^  = 0.70±0.004). Current SII of these two allopatric strains is slightly higher but similar to the index obtained in earlier assays (0.61±0.07, in [37]). In contrast, sexual isolation of present-day mating choice assays between untreated AM and CA semispeciesis (assay 10, SII_AM_^U^_X__CA_^U^  = 0.90±0.002) is significantly stronger (*P* = 0.0164) than observed in earlier assays in the mid 1960s (0.70±0.07, in [37]).

Assays performed on AM and OR semispecies, where both partners were treated (AM^T^ x OR^T^), resulted in a weak enhancement of SII from 0.70±0.004 in both untreated to 0.86±0.002 and 0.84±0.003 under low- and high-Rif conditions, respectively (assays 1 to 3). However, this slight effect on sexual isolation is not statistically significant under both treatment conditions (*z* = 1.88 for 0.01% Rif and 1.41 for 0.1% Rif, both *P*>0.05). Under 0.2% Rifampicin, however, double treatment of both partners induced almost random mating (assay 4; and Table S2, *z* = 3.89, *P*<0.0001).

Unidirectional selective treatment of OR semispecies at low-Rif conditions slightly reduced sexual isolation when OR was mated with untreated AM semispecies, from SII_both untreated_  = 0.70±0.004 to SII_AM_^U^_X OR_^T^  = 0.54±0.004 (assay 5), although this reduction is not statistically significant (*z* = 0.18, *P*>0.05). More intense treatments of OR semispecies with 0.1% Rifampicin, however, revealed a 33% reduction of assortative matings (assay 6), compared to both untreated controls (SII_AM_^U^_X OR_^T^  = 0.48±0.007 *vs*. SII_AM_^U^_X__OR_^U^  = 0.70±0.004). Although increased dosages of antibiotics in OR semispecies further reduce sexual isolation in AM^U^ x OR^T^ assays, *Fisher's Exact* tests and *t* tests show only weak statistical significance (*z* = 1.93; *P* = 0.0539). Similar to double treatment assays (assays 2–4), in OR semispecies higher doses of Rifampicin up to 0.2% were necessary to significantly reduce sexual isolation (SII  = 0.33±0.007) in heterogamic mating choice assays (assay 7; Table S2, *z* = 3.19, *P*<0.0014).

In contrast to OR harboring *Wolbachia* at high-titer, selective antibiotic treatments of low- titer AM semispecies trigger dramatic effects on assortative matings in AM^T^ x OR^U^ mating choice assays (assays 8 and 9). Combinations between AM treated and OR untreated lines resulted in the complete breakdown of sexual isolation towards random mating between these two *D. paulistorum* semispecies. Random mating was observed in combinations of AM^T^ x OR^U^ under conditions of low- (0.06±0.008) and high Rifampicin (0.18±0.008), where, in both cases, reductions were statistically highly significant (for both treatment conditions: *z* = 3.89, *P*<0.0001).

Combinations between the two *Wolbachia* low-titer semispecies CA and AM (assays 10–13) showed that partial depletion of the symbiont via 0.1% Rifampicin decreases levels of mate discrimination by about 50% in all three directions, *i.e.* in double and both unilateral treatments. SII dropped from an original 0.90±0.002 (both untreated) to 0.38±0.007 (AM^T^ x CA^T^), 0.46±0.007 (AM^T^ x CA^U^), and 0.44±0.007 (AM^U^ x CA^T^), respectively. For all three combinations reductions in mate discrimination were highly significant (*z* = 3.89, *P*<0.0001).

Finally we have assayed the effect of partial *Wolbachia* depletion on assortative mating behavior in our most recently collected *D. paulistorum* samples belonging to AB semispecies, *i.e*., high-titer POA1 and low-titer POA10 lines (Figure S3). Mating choice assays were performed in heterogametic combinations between untreated CA semispecies and both POA lines under high Rifampicin treatment conditions (Figure 5, assays 14–17). Similar to “old” laboratory strains of AM, CA and OR semispecies, both AB lines obtained from more recent collections showed statistically significant reduction in their assortative mating behavior after partial symbiont depletion in combinations with untreated CA semispecies (Table S2, *z* = 3.89, *P*<0.0001). SII dropped from original 0.92±0.001 (POA1 in assay 14), and 0.95±0.001 (POA10 in assay 16) to 0.49±0.006 (assay 15), and to 0.35±0.007 (assay 17), respectively.

In order to rule out potential toxic side effects of antibiotics on Drosophila mating behavior and sexual isolation, we have generated symbiont-depleted lines of the *D. simulans* Riverside strain naturally infected with *w*Ri *Wolbachia* [60]. 0.2% Rifampicin treatments were performed for five consecutive generations in accordance to our depletion protocol for *D. paulistorum* lines (Materials and Methods). Standard mating choice assays were performed between untreated (*D. sim* ^U^) and treated (*D. sim* ^T^) samples. As shown in Figure 5, high doses of Rifampicin have no effect on random mating behavior in native *D. simulans*-*Wolbachia* associations (assay 18; and Table S2). These data implicate that loss of assortative mating behavior in *D. paulistorum* semispecies was most likely caused by disturbing natural host-symbiont associations, rather than by any unspecific toxic drug-effect.

In addition there is a direct correlation between the natural *Wolbachia* titer level in a defined semispecies and their sensitivity to alterations in assortative mating behavior upon partial symbiont depletion. OR semispecies, harboring high-titer levels of their core-endosymbiont, seem more recalcitrant to antibiotic treatments than AM and CA. Similar to the sex ratio distortion phenotype (Table 1), OR semsipecies need higher dosages of antibiotics (up to 0.2% Rifampicin) in order to reach the critical threshold level sufficient for triggering statistically significant changes of mating behavior. A similar but statistically not significant correlation between natural symbiont titer level and drug competence was observed in POA lines belonging to AB semispecies. Whereas the high-titer line POA1 showed a moderate 46.7% reduction in mate avoidance after partial symbiont depletion, in the low-titer POA10 line a 63.2% reduction was observed (Figure 5, assays 14–17).

**Figure 6:**
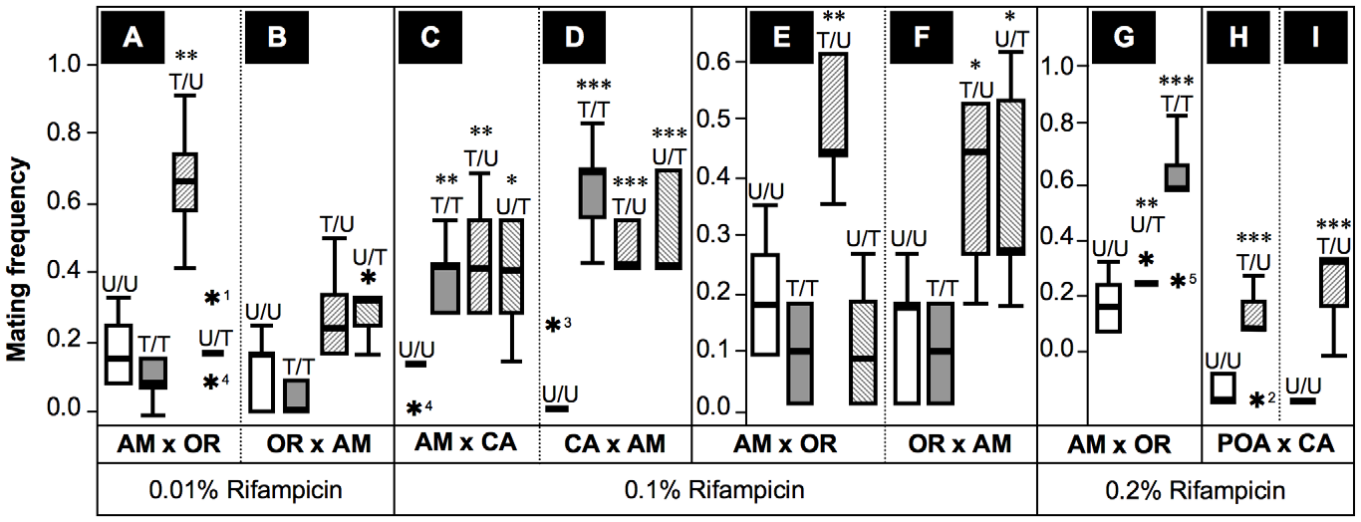
Frequencies of successful heterogamic matings of *D. paulistorum* females. Box plots represent distribution of mating frequencies obtained from interstrain mating choice assays with Amazonian (AM), Centroamerican (CA), Orinocan (OR) and Andean-Brazilian (AB; lines POA1 and POA10, respectively) semispecies (females are first named). Tested lines were kept on normal food, or on 0.01% (**A**,**B**), 0.1% (C–F) and 0.2% Rifampicin (**G–I**). Mating frequency was determined by number of successful heterogamic matings out of twelve females in five replicas each (Table S2). Combinations of heterogamic mating choice pairs are indicated by U/U  =  both partners untreated; T/T  =  both treated; T/U  =  females treated, males untreated; and U/T  =  females untreated, males treated. Statistical significant values are indicated by one, two or three asterisks, *i.e*., *P*<0.05, *P*<0.01 and *P*<0.001, respectively; and statistical outliers by black stars.

Summing up, the mating choice assays presented here imply strong effects of *Wolbachia* on premating isolation between *D. paulistorum* semispecies in a titer-dependent manner. Next we asked the question as to whether artificial symbiont depletion modifies mate avoidance patterns on the part of *D. paulistorum* females. For that purpose we have determined female mating frequencies of successful heterogamic matings obtained from mating choice assays itemized in Figure 5 and Table S2. As shown in Figure 6, untreated females of AB, AM, CA and OR semispecies strongly oppose heterogamic males, resulting in rare heterogamic mating events under our assay conditions. Under low-Rif conditions (0.01%) only AM-treated females lose mate discrimination by accepting heterogamic OR males (Figure 6A), whereas no significant alterations were observed in treated high-titer OR females (Figure 6B).

Under 0.1%-Rif conditions, however, treated AM and CA females (Figure 6C–6E) accepted reciprocal heterogamic males of untreated (T/U) and treated origins (T/T) significantly more often than do untreated females (*P*<0.01). In addition, treated OR females mate with untreated AM males (T/U) significantly more frequently (*P*<0.05) than do untreated females (Figure 6F); higher concentrations of the antibiotic (0.2%) further enhanced effects on mate recognition patterns of OR females in a dose-dependent manner (Figure 6G). Similar to the “old” laboratory strains mentioned above, treated females of the two more recently established AB lines showed similar behavioral patterns (Figure 6H,6I). To sum up, our data imply that partial depletions of *Wolbachia* in females of AB, AM, CA and OR semispecies significantly impair their mate avoidance behavior; they lose proper mate recognition signals and finally accept improper heterogamic males.

Besides this effect observed in treated females, partial *Wolbachia*-depletion also affects male behavior. As shown in Figure 6C,6D treated AM and CA males succeed in copulating significantly more frequently with untreated heterogamic females (U/T) than do untreated males. This enhanced mating frequency on the part of treated males is statistically significant (*P*<0.05 for CA and *P*<0.001 for AM males, respectively). Enhanced mating success of treated AM males (*P*<0.05) was also observed in combinations with untreated OR females (Figure 6F), suggesting that partial symbiont-depletion is capable of also changing male sexual behavior in both low-titer semispecies. Our current Rifampicin conditions up to 0.2% applied to OR semispecies, however, were not sufficient to induce a similar change in male mating behavior, since mating success of treated OR males (U/T) equals the one observed for untreated males (U/U) in heterogamic AM assays (Figure 6E,6G). These data suggest that even our current 0.2% treatment conditions are not sufficient for altering mating behavior of OR males that naturally harbor high-titer *Wolbachia*.

## Discussion

### Uncovering Hidden *Wolbachia* Diversity in *D. paulistorum* Semispecies

The presence of low-titer *Wolbachia* in five out of the six *D. paulistorum* semispecies corroborates earlier ultrastructural observations, showing that “*MLO*s” reside at low densities in the reproductive organs of their natural hosts, but heavily overreplicate in hybrids [49]. This quantitative shift of *Wolbachia* densities from extremely low in native hosts to intermediate in interstrain hybrids, strongly suggests that the causative agent of incipient *D. paulistorum* speciation are *Wolbachia*. Moreover no other germ line-associated microbes were isolated by different means of experimental 16S consensus PCR approaches.

In adult flies of AB, AM, CA, IN and TR semispecies, densities of native endosymbionts are too low for unambiguous detection by standard *wsp* PCR protocols. As such, earlier attempts have failed to isolate *Wolbachia* from members of the *D. paulistorum* species complex and hence were diagnosed as *Wolbachia*-free [59], [73]. Importantly, none of these earlier studies reported surveys neither of the high-titer OR semispecies, nor of F1 interstrain hybrids, where *Wolbachia* is ostensible. In a recent publication we have applied our PCR-*wsp*-hybridization strategy [53] to uncovering multiple low-titer *Wolbachia* strains in the cherry fruit fly, *Rhagoletis cerasi*, not accessible by standard PCR methods in earlier studies. We hence encourage considering the broader existence of low-titer *Wolbachia* in nature, commonly escaping conventional detection approaches, before designating a sample or even a whole group under study as uninfected.

Based on *wsp* and *VNTR* sequence analyses, *Wolbachia* of CA, OR and TR semispecies are host-specific and are closely related with, but not identical to, other *w*Au-like strains isolated from phylogenetically germane neotropical *Drosophila* hosts [59] that harbor unique diagnostic sites (Table S1). As shown in Figure S3, apparent conspecificity between native hosts and defined *Wolbachia* strains of *D. paulistorum* semispecies and their related sister species suggests an ancestral association assisted by stable vertical transmission. In order to elucidate the coevolutionary history of this host-symbiont interaction, more detailed molecular *Wolbachia*-strain-typing analysis will be necessary for *D. paulistorum* semispecies, from strains kept since the 1950s in the laboratory, as well as from recent samples of natural populations, by means of applying more informative, preferentially non-coding *Wolbachia* markers other than the *wsp* locus or other protein coding genes. As such, there are a growing number of cases in recent *Wolbachia* literature, where strains sharing identical *wsp* sequences induce clearly distinguishable phenotypes [74]–[77]. Importantly, these reported cases of phenotypic transitions of *Wolbachia* in fruit flies, aphids and butterflies, took place in extremely short evolutionary time scales, implying that these symbiotic associations are highly dynamic and rapidly evolving in natural and laboratory populations.

In contrast to the *w*Au-like *Wolbachia* of CA, OR and TR, the core-endosymbiont of AM semispecies is unique in that it groups closely with the *w*Ri infection of *D. simulans*, suggesting a more recent acquisition via horizontal transmission from a yet uncharacterized external source. Potentially, this new *Wolbachia* strain has replaced an ancestral *w*Au-like symbiont, similar to CA, OR and TR *Wolbachia*, already present in the AM progenitor lineage. The uniqueness of *w*Ri-like *Wolbachia* in AM corroborates with earlier phylogenetic studies, designating AM as the most distal semispecies of the *D. paulistorum* complex [78], [79].

### Compensatory Evolution between *Wolbachia* and Their Natural *D. paulistorum* Hosts

In agreement with earlier reports, *D. paulistorum* semispecies are highly sensitive to antibiotics [41]. In addition, ultrastructural analyses have shown that no *D. paulistorum* fly has ever been observed that is devoid of endosymbionts [39], [41], [49]. Since complete clearance of *Wolbachia* is obviously lethal for all *D. paulistorum* semispecies, it has been suggested that core-endosymbionts of *D. paulistorum* are well-adapted obligate mutualists that serve essential vital functions benefiting to their hosts (reviewed in [42], [65]). In this study we note that partial symbiont depletion under mild antibiotic condition is capable of triggering alterations in (*i*) female fecundity, (*ii*) male-biased sex ratio, and (*iii*) most significantly, female mating behavior in *D. paulistorum*.

The female fecundity phenotype, induced by artificial depletion of *Wolbachia* (Figure 4), engenders similar effects on oogenesis that have been detected in the parasitoid wasp *Asobara tabida*, where *Wolbachia* is an obligate symbiotic partner [80], [81]. Female wasps that are cured of their *Wolbachia*, fail to produce any mature oocytes, rendering *Wolbachia* infection essential for female reproduction. In this case of mutualism, *Wolbachia* influence programmed cell death processes in *A. tabida* by modulating apoptosis during oogenesis [15]. The authors speculate that after a loss-of-function mutation in a key regulator gene of the *A. tabida* apoptosis network system, *Wolbachia* were capable of permanently taking over control of the host apoptotic pathway. Another example of the compensatory effect of *Wolbachia* on a host loss-of-function mutation, comes from laboratory strains of *D. melanogaster*, where the presence of *Wolbachia* is sufficient to restore the ovarian phenotype in *Sxl^f4^* mutants [66]. The most recent example showing that *Wolbachia* can transform even in an obligate nutritional mutualist residing in a bacteriome was found in the bedbug *Cimex lectularius*, where artificial depletion of its symbiont results in sterility and retarded growth [82].

The second phenotype, uncovered by mild antibiotic *D. paulistorum* treatments (Table 1), is, to our knowledge, the first report on a symbiont-induced male-biased sex ratio distortion in arthropods. In filarial nematodes, significant male-biased sex ratio distortion was observed after partial depletion of mutualistic *Wolbachia* by Tetracycline [67], [68]. These authors suggest that if *Wolbachia* play a more active role in females than in males, this may imply a more biosynthetic activity of *Wolbachia* in female hosts and thus greater susceptibility to antibiotics. This assumption is based on evolutionary models [83], [84], asserting that in a mutualistic relationship, selection on inherited microorganisms should (*i*) select directly for beneficial effects towards the host sex responsible for their transmission (in general, the female), and (*ii*) favor the spread of phenotypes that are detrimental to those hosts not involved in their transmission (males or uninfected females). In this respect, the profound sensitivity of *D. paulistorum* females to *Wolbachia*-depletion (loss-of-function) plus the hybrid male sterility phenotype caused by *Wolbachia* overreplication in testes (gain-of-function) corroborates this evolutionary model.

Both phenotypes, low female fecundity and male-biased sex ratio distortions, induced by mild antibiotic treatments of *D. paulistorum* semispecies (Figure 4 and Table 1), can be explained by strong obligate mutualistic interactions between *Wolbachia* and their natural neotropical hosts, *i.e*., suppression of enhanced cellular mortality during female development or a direct effect on the Insulin/IGF-like signaling cascade.

The first model assumes that the presence of *Wolbachia* above a specific threshold level plays an essential role in female, but not male, development by suppressing female lethality. For example, female larvae or pupae require a nutrient vitamin or hormone contributed by *Wolbachia* in order to develop properly and to mature gametes. In addition, immune responses of a host against α-proteobacteria frequently involve apoptosis of infected cells, and, in turn, symbionts can inhibit host cell suicide to circumvent the immune system [85]. As recently shown, the *Wolbachia*-encoded surface protein WSP is capable of inhibiting apoptosis *in vitro* [86]. Hence the permanent association between *Wolbachia* and *D. paulistorum* hosts is likely to be the result of a tight coevolution between an ancestral host responding to infection with apoptosis, the symbiont suppressing it, and the host compensating for this suppression because apoptosis is required for development.

Alternatively but not exclusively, depletion of *Wolbachia* in *D. paulistorum* semispecies by mild antibiotic treatment might have unleashed a cryptic, but female-biased loss of function phenotype associated with the Insulin/IGF-like signaling (IIS) pathway. Mutations that reduce the activity of the IIS signaling cascade have pleiotropic effects on many traits, such as development, metabolic homeostasis, adult lifespan and fecundity in nematode worms, fruit flies, and mice (reviewed by [87]). As was recently shown, removal of symbiotic *Wolbachia* in naturally infected *D. melanogaster* reduces IIS and hence enhances mutant IIS phenotypes, suggesting that *Wolbachia* normally act to increase insulin signaling [88]. Since some of the key downstream effectors of the IIS cascade are also functionally implicated in regulating apoptosis [89], [90], one may assume that these two models explaining the evolution of compensatory mutualistic *Wolbachia* in *D. paulistorum* semispecies, are not mutually exclusive. Under both evolutionary scenarios, however, closely related but host-specific *Wolbachia* strains might act as general suppressors of yet undefined, cryptic, female-biased mutations already present in the common ancestor of all the *D. paulistorum* superspecies. This assumption is corroborated by our findings that at least three out of the six infected semispecies (CA, OR and TR), from which we have already obtained sufficient information on *Wolbachia*-strain diagnostics, harbor strain-specific and closely related *w*Au-like endosymbionts (Table S1). In addition, the TR semispecies is regarded as the relict ancestral semispecies of the *D. paulistorum* superspecies [78], [79] further supporting their long term relationship and common ancestry with *w*Au-like *Wolbachia* symbionts.

As such, regulation of host-programmed cell death during female development, as well as suppression of a cryptic IIS mutation in *D. paulistorum* semispecies, are both plausible mechanisms that can explain the evolutionary transition of *Wolbachia* from facultative parasitism towards obligate mutualism via compensatory evolution. Under both models, *D. paulistorum Wolbachia* can be regarded as obligate mutualists by compensating reduced female-specific fitness effects that were originally caused, either by a suppressor of IIS mutation, or by reproductive parasites such as *Wolbachia*. In each of the two scenarios, a former reproductive parasite became domesticated by its host system, thereby providing more recently evolved immunity against genomic loss of function mutations - and intracellular parasites, respectively. In summary, the coevolutionary interaction between *D. paulistorum* semispecies and *Wolbachia* infection has changed from antagonistic into reciprocally beneficial and thereby into an obligate mutualistic interaction, where the both systems are dependent on each other now.

### The Impact of *Wolbachia* on Infectious Speciation

Increasing empirical evidence and numerous theoretical models predict that transovarialy transmitted microbial symbionts can have significant impacts as drivers of speciation processes in their natural hosts [12], [17]–[21]. However, there are substantial difficulties with speciation based solely on symbionts capable of inducing incompatibilities [1], [22]. First, as deduced from theoretical models, CI causes unstable equilibria in prevalence and transmission rate of symbiontic infections [16], [26], [27], but more recent models show that such stabilities can exist, depending on parameters such as host migration rate, CI induced gene flow reduction and the invasion success of mutants at a mate preference locus [21]. Moreover, this criticism has been refuted in Bordenstein 2003 and is not *Wolbachia*-specific since it applies to any type of postzygotic incompatibilty [12]. In our *Wolbachia-D. paulistorum* model system however, theoretical caveats opposing most speciation models can be overruled by evolving ancestrally fixed, obligate mutualistic interrelations between the endosymbiont and its natural host, vital for both partners. So, even partial titer depletion of the obligate core symbiont below a critical *Wolbachia* threshold, results in severe pleiotropic and primarily female-biased phenotypes, which natural selection acts against.

Second, the few empirical systems from present literature concerning roles of symbionts in speciation, such as parasitoid wasps of the *Nasonia* sibling species group, are highly informative in elucidating the evolutionary implications of endosymbionts [28], [29]. Bordenstein and colleagues showed that in the youngest *Nasonia* species pair, *N. giraulti* and *N. longicornis*, *Wolbachia*-induced bidirectional CI was the primary form of reproductive isolation prior to the evolution of other isolating barriers. However, these species are not known to occur sympatrically in nature and premating isolation tested in the laboratory was weak and asymmetric [12], [29]. Hence it has been suggested to focus on natural symbiotic systems of sympatric, hybridizing host species that are isolated because of CI [12]. Indeed, the mushroom feeding Drosophila species *D. recens* and *D. succinea* are found in a hybrid zone in central Canada. Moreover they express strong unidirectional CI triggered by *Wolbachia* in *D. recens*, plus asymmetrical premating isolation and hybrid male sterility. The latter two isolation mechanisms, however, are genetically based [20], [30]. The assortment of symbiotic and genetically based isolation barriers provides clear evidence that *Wolbachia* can act in concert with genetic and/or geographic isolation mechanisms in nature. Our incipient speciation system presented here is in flagrant *statu nascendi*, occurs sympatrically in consistently overlapping geographic distributions in Middle and South America [43], exerts strong bidirectional CI [44]–[46], plus pronounced premating isolation in a symbiont-dependent manner (this study).

Finally, no empirical cases have been reported suggesting that endosymbionts are able to cause hybrid-male sterility, a hallmark of an early stage in the evolution of postzygotic isolation [1], but also *see* [32], and this study. However, the novel *Wolbachia*-induced phenotype of symbiont-induced hybrid male sterility reported here, can be regarded as currently residual and supportive, since all *D. paulistorum* semispecies have evolved strong prezygotic isolation mechanisms and intriguing female mate avoidance patterns by successfully preventing inter-semispecific matings [37]. Furthermore, natural hybrids between sympatric semispecies have never been reported in the field, nor has a trapped sperm-storing gravid female ever borne alien sperm (L.E. personal communication).

After having demonstrated that *Wolbachia* are ancestrally fixed, obligate entities of all *D. paulistorum* semispecies, perfectly transmitted by the mother and vital for female oogenesis and female development, we have evaluated effects of mild symbiont depletion via antibiotics on female mating choice. Currently a growing number of reports emerge in our literature comparing specific behavioral traits possessed by symbiont-infected and uninfected individuals, such as mate avoidance, male promiscuity, longevity, pathogenic fungal resistance, and olfactory-cued locomotion [31], [91]–[95]. In spider mites, for just one example, *Wolbachia*-associated unidirectional CI can be avoided by females at the premating level [91]; they show spider-mite females exhibiting both precopulatory and ovipositional behaviors that increase odds of successful compatible matings. Furthermore, in mating choice experiments, uninfected females preferably mate with uninfected males, and in doing so, directly reduce opportunities for *Wolbachia*-induced unidirectional CI expression [91].

So according to the scenario observed in spider mites, we will propose the following model for understanding the biological role of *Wolbachia* driving sexual isolation between *D. paulistorum* semispecies: Obligate mutualistic *Wolbachia*, having evolved tight compensatory interrelations with their natural hosts, are capable of triggering female mate recognition patterns in favor of preferentially accepting males harboring the same type of *Wolbachia - i.e*., members of their natal semispecies - and to reject improper mates carrying an alien version of their obligate symbiont. In the mixed genetic background of artificially generated hybrids however, maternally transmitted, naturally benign mutualistic *Wolbachia* turn into reproductive parasites, capable of inducing strong embryonic bidirectional CI and complete male hybrid sterility via loss of their controlled replication. Although F1 hybrid females are fertile under laboratory conditions, they show an intriguingly altered sexual “old maid” behavior by hardly accepting any kind of mating partner [96]. Hence we assume that symbiont-directed mate recognition could have evolved in order to prevent strong bidirectional CI and reduced sexual success of potential hybrids, thereby ensuring their continuing vertical transmission. Alternatively, native hosts might have evolved behavioral avoidance patterns that recognize potential mates carrying distinctive but incompatible *Wolbachia* variants [91]. As suggested by Koukou and colleagues *Wolbachia* might have evolved the capacity to modulate host pheromone expression and/or perception [31]. Recently, Albertson and colleagues [97] have shown that *Wolbachia* significantly concentrate in specific brain regions (*i.e*., the subesophageal ganglia, superior protocerebra and antennal lobe) of *D. melanogaster* and *D. simulans*; speculating that the presence of the endosymbiont could potentially affect insect behavior, such as olfactory-cued locomotion and mating behavior [95]. By analytical organic methods it was recently discovered that within the *D. paulistorum* complex the active male and female pheromonal compounds differ in proportions present in each of the six *D. paulistorum* semispecies - that is, quantitatively not qualitatively [79], [98]. In accordance with theses data, we revealed that *Wolbachia* are also tightly associated with the antennal lobe of *D. paulistorum* semispecies (unpublished data). Hence, our *D. paulistorum-Wolbachia* symbiosis system, affecting premating isolation between natural semispecies, provides a unique and excellent model system for studying the global impact of microbial endosymbionts on pheromone production, olfaction, and sexual behavior in insects.

## Materials and Methods

### Fly Strains

The *D. paulistorum* species complex [99] is neotropical and comprises at least six semispecies in *statu nascendi* [35]. They are morphologically indistinguishable, but can be identified by allozymes, chromosomes and courtship behavior [42]. For this study, the following four strains, belonging to Amazonian (AM), Centroamerican (CA), Orinocan (OR), and Transitional (TR) *D. paulistorum* semispecies were used: Belem, Brazil, AM; Lancetilla, Honduras, CA; Georgetown, Guyana, OR; and Santa Marta, Colombia, TR. For full descriptions of origins and maintenance of all *Drosophila paulistorum* strains, *see* [42], and references therein. POA1 and POA10 strains, belonging to the Andean Brazilian (AB) semispecies are isofemale lines that were generated from collections in April 2003 in Porto Alegre City, Rio Grande do Sul State Brazil. Both lines were kindly provided by Yong-Kyu Kim, Emory University, Atlanta, GA, USA. The control strain Pan98, collected in Panama in 1998, belongs to the closely related sister species *D. willistoni*, naturally infected with *w*Wil *Wolbachia* [59]; and two *D. simulans* lines, Riverside (RI) infected with *w*Ri *Wolbachia* [60] plus the uninfected strain STC [100] serve as positive and negative controls, respectively.

### Generation of *D. paulistorum* Interspecies Hybrids

Sexually mature virgin females of AB, AM, CA and TR semispecies were forced to mated *en mass* with a twofold excess of heterogamic OR males, respectively. These males had been isolated from females for several days. Flies were transferred into fresh vials every 48 hrs over a period of two weeks.

### Molecular Isolation and Strain Typing of *D. paulistorum* Semispecies *Wolbachia*

High quality genomic DNA was extracted from a pool of ten adult flies, 15 ovaries/testes or from 30–50 mg embryos using the QIAamp DNA Mini Kit (Qiagen, Hilden, Germany). Eggs were collected from apple juice agar plates in 24 hrs intervals and sterilized by extensive washes with 70% EtOH before DNA extraction. Testes and ovaries were dissected in sterile 1x PBS. All DNA samples were stored at −20°C until use.

### Germline-Associated 16S rRNA PCR Screen

In order to determine the microbial diversity of germline associated symbionts of *D. paulistorum* OR semispecies, we have performed 16S rRNA consensus PCRs after [50] on five independent DNA samples, derived from sterilized 0–24 hrs embryo. From each sample, at least four amplified 16S rRNA consensus fragments were cloned and sequenced. All twenty sequence-reads were identical with the 16S rRNA gene of *Wolbachia pipiens* of *D. willistoni* (accession number DQ412086), and no other bacterial sequence was detected. To further confirm the presence of *Wolbachia* in reproductive organs of *D. paulistorum* we performed two independent 16S rRNA universal PCRs after [50], [51] on sterile-dissected ovaries and testes of AM, OR semispecies and AxO hybrids derived from crossings of AM females with OR males. Two different primer sets amplifying overlapping universal microbial 16S rRNA fragments between 0.4 and 0.6 kb were used. Amplicons were purified using the Promega Wizard SV Gel PCR Clean-up system. Sequences were obtained via cycle sequencing with BigDye Terminator v3.1 performed at the Department of Marine Biology, University of Vienna, Austria. Sequences were analyzed using ApE plasmid editor v1.10.4 (M. Wayne Davis), ClustalX, (www.clustal.org), Mesquite v2.6 (www.mesquiteproject.org), and the BLAST algorithm (www.ncbi.nlm.nih.gov).

### Diagnostic *Wolbachia* PCR, Cloning and Sequencing

*w*Pau *Wolbachia* was detected using primer specifically targeting the *wsp* locus, the transposon *IS*5 as well as *VNTR*-105 and *VNTR*-141 locus. *wsp*-primer sets were taken from [52] and primers for VNTRs and *IS*5 PCR were taken from [57]. In all reactions the uninfected *D. simulans* strain STC was included as negative controls. Diagnostic *wsp*-PCR reactions were performed under the following conditions: 10 µl reactions containing 1 µl of genomic DNA template in 1x polymerase reaction buffer (Promega, Madison, USA), 4.5 mM MgCl_2_, 0,3 µM of each primer, 150 µM of each dNTP and 0.4 U of *Taq* polymerase (Promega, Madison, USA). Diagnostic VNTR-PCRs were performed as follows: 10 µl reactions containing 1 µl of genomic DNA template in 1x polymerase reaction buffer (Promega, Madison, USA), 2,5 and 4 mM MgCl_2_ for *VNTR*-141 and −105, respectively, 0,3 µM of each primer, 35 µM of each dNTP and 0.4 U of *Taq* polymerase (Promega, Madison, USA). PCR-profiles for *wsp* amplifications were taken from [52]. For *VNTR*-141 locus, amplifications consisted of 5 min at 94°C followed by 34 cycles of 1 min at 94°C, 1 min at 50°C and 1 min 30 sec at 72°C. Final elongation step was performed at 74°C for 8 min. For *VNTR*-105 a higher annealing temperature of 61°C was used and elongation was performed for 3 min instead of 1 min 30 sec. *IS*5 PCRs were applied as described in [55]. For all PCR reactions a Biometra T3000 Thermocycler (Biometra, Goettingen, Germany) was used. PCR products were purified using the peqGOLD Gel Extraction Kit (peqLab, Erlangen, Germany). For cloning, products were inserted into the pTZ57R/T vector (Fermentas, St. Leon-Rot, Germany), and used to transform competent DH5α *Escherichia coli* cells. Clones containing the insert were cycle sequenced with BigDye Terminator v3.1 at the Department of Marine Biology, University of Vienna, Austria. Sequences were analyzed as described above.

### Detection of Low-Titer *Wolbachia* via Southern Hybridization

In order to detect low-titer *Wolbachia* infections in *D. paulistorum* semispecies, a digoxigenin (DIG, Roche Diagnostics, Germany) labeled probe, binding to the core sequence of the *wsp* gene, was applied on blots of standard *wsp* PCR [52] for DNA-DNA hybridization. For probe design, hybridization conditions and signal detection see our detailed protocol in [53].

### Antibiotic Treatment Conditions

Stock solutions of antibiotics were made by diluting Tetracycline (3 mg/ml) and Rifampicin (30 mg/ml) into ethanol 98%, stored at −20°C. Artificially *Wolbachia*-depleted strains of AB, AM, CA and OR *D. paulistorum* semispecies were established by adding aliquots of the respective stocks to regular fly food at final concentrations of 0.01 and 0.03% of Tetracycline and 0.01, 0.03, 0.1 and 0.2% of Rifampicin. Treated lines were kept for at least three generations on antibiotics for bioassays.

### Ovary Phenotype Assay

Analysis of ovary phenotype followed [66]. Females were raised on standard food with or without antibiotics (0.01% Tetracycline for fifteen generations and 0.1% Rifampicin for three generations) at 25°C and were dissected ten days after eclosion. Only eggs reaching stage 13 (after [101], indicated by filaments) were counted as mature eggs. For DAPI staining, ovaries were dissected ten days after eclosion in sterile *Drosophila* Ringer's Solution. Ovaries were stained in a DAPI-TBST solution (1 mg/l) for 5 minutes under constant agitation and mounted in Citifluor Glycerol/PBS solution (Gröpl, Austria). Ovaries were analyzed using a Nicon Eclipse E800 fluorescent microscope.

### Immunohistochemistry

*Wolbachia* density and tissue tropism in *D. paulistorum* semispecies were determined using the polyclonal WSP (Wolbachia surface protein) antibody [102]. WSP protein expression was analyzed in whole mount immunostainings on ovaries, staged embryos and on sections of male tissues after [103]. Rabbit anti-*wsp* antibody was used at a 1∶500 dilution overnight at 4°C and detected after incubation with a 1∶500 dilution of Alexa Fluor 488 goat anti-rabbit IgG secondary antibody (Molecular Probes) at room temperature for 1 hour. Slides were stained for 3 min with 1 µg/ml DAPI (Molecular Probes), rinsed and stained with 5 µg/ml propidium iodide (PI) (Molecular Probes) for 20 minutes, rinsed again and mounted with ProLong Antifade medium (Molecular Probes). Immunostainings of embryos and ovaries were examined by using a Zeiss Axiomot 2 Epifluorescence microscope. Images were processed using Photoshop 9.0.2 (Adobe).

### Sex Ratio Assays

Sex ratio in the *D. paulistorum* semispecies Amazonian (AM) and Orinocan (OR) emerging from regular food (untreated) and two independent antibiotic-treatments with teracycline (Tet) and Rifampicin (Rif). Total number of flies counted for two weeks from initial day of hatching was 8,693 divided into 4,851 males and 3,842 females. Untreated AM and OR semispecies have the usual 1∶1 sex ratio (also *see* [42]; and references therein). Both antibiotic treatments were performed either over five generations on 0.01% Tetracycline (Tet) or 0.01% Rifampicin (Rif); or over three generations on 0.03% and 0.1% Rifampicin (Rif).

### Measuring Sexual Isolation

Mating choice assays, monitoring the effect of artificial symbiont depletion via Rifampicin at 0.01% and 0.1% for at least five generations, involved direct mating observations. All details concerning our experimental protocols have been itemized by [37], and reviewed and adjudicated by [104]. Briefly: All replicas were conducted mornings at room temperature in daylight facing north. Beforehand, virgin flies were aged two to three days after light ether anesthetization during which they were sexed (there are no sex differences in abdominal banding in this superspecies); and half were marked via minute distal wing clips (controlled in bioassays). These marks were rotated (wing to wing and treated to untreated). Such abrasions have tested neutral regarding behavioral influences [105] in this superspecies.

For each of the eighteen interstrain mating choice assays monitoring the impact of Rifampicin treatment on mate avoidance between AB, AM, CA and OR semispecies and *D. simulans* controls, five replicas and 120 matings were scored (12A ♀♀ +12B ♀♀ +12A ♂♂ +12B ♂♂ differentiated by rotated wing clips) totaling 2,160 matings. Without repeated anesthetization at any time, 2-to-3-day old flies were then placed into glass mating chambers [37], [106]. A four-power hand lens was employed for scoring partners: the time (from start of observations) each mating takes place; its sequence among other copulae which occur; where in the chamber the mating pair is located (for this purpose a grid constitutes the floor); the kind of female involved; and the kind of male involved, until all flies copulated, in approximately 30–40 minutes. If undisturbed, each copulation lasts approximately 15–17 minutes; females mate only once in these arenas. Recording location of copula, even upside down, prevented scoring a copula more than once.

Sexual isolation index (SII) and standard error (SE) were measured as in previous studies using multiple mating choice assays, following two calculations, *i.e.*, the isolation index after [72] and the joint isolation index of [107]. In our assays, both calculations result in very similar SII values (Table S2). In general, SIIs range from −1.00 (preference for unlikes, heterogamy) through 0 (random mating) to +1.00 (preference for likes, homogamy) between *D. paulistorum* semispecies.

### Statistical Testing

All statistical tests were performed using SPSS 16.0 for Mac and GraphPad Software (www.graphpad.com). Statistical significance of effects of antibiotic treatment on sex ratios in *D. paulistorum* semispecies was tested calculating single degree-of-freedom Chi-squares. We applied an unpaired *t*-test to mating frequencies in *D. paulistorum* combinations, and compared the SIIs obtained from mating choice assays using *Fisher's Exact* test. Boxplot diagrams of mating frequency distributions were generated using SPSS.

### Detection of *Wolbachia* in *D. paulistorum* Hosts via Transmission Electron Microscopy

To determine presence of *Wolbachia* within *D. paulistorum* hosts, living adult flies were sectioned using a vibrating blade microtome (Leica VT1000 S). Sections of 400–500 µm thickness were fixed in 2% glutaraldehyde, buffered with 0.1 M PO_4_ at pH 7.1. After postfixation with 1% OsO_4_, samples were dehydrated via an ethanol series [108]. Samples were then embedded in Epon and 70 nm ultrathin sections were produced. Following uranyl acetate and lead citrate staining of the sections, presence of *Wolbachia* was observed using a JEOL TEM.

### Accession Numbers

Sequences were deposited in GenBank under the accession numbers GQ924884-GQ924891 and HQ185362-63.

## Supporting Information

Figure S1 *Wolbachia* infection in adults of *Drosophila paulistorum* semispecies. Amazonian (AM); Centroamerican (CA); Interior (IN); Andean-Brazilian (AB); Transitional (TR); Orinocan (OR) semispecies and one F1 hybrid derived from crossings of CA females to OR males (CxO). (**A**) *wsp*-long PCR and (**B**) blot followed by hybridization with DIG-labeled *wsp*-probe (*see* Materials and Methods).
(0.53 MB TIF)

Figure S2 Co-cladogenesis of *Wolbachia* and host. Host tree (left) was calculated from nine *Adh* sequences of Drosophila species (AY364506; X57362; AE014134.5; AF045118; DWU95252; EU532128; EU532123; EU532127; EU532121) with 405 sites including gaps; method  =  average linkage (UPGMA). *Wolbachia* tree (right) was generated from nine *wsp* sequences (AY897491; AF020070; GQ924887; AY620227; AF620218; AF020063; GQ924889-90) with 652 sites including gaps; same method. Alignments (L-INS-i strategy) and tree calculation were performed using MAFFT 6.0 (http://mafft.cbrc.jp/alignment/server/); trees were edited using FigTreev1.2 (http://tree.bio.ed.ac.uk/software/figtree/). Asterisk indicates a region of rough resolution; for high resolution *see* SNP analysis in Table S1.
(0.19 MB TIF)

Figure S3 Natural presence of high- and low-titer *Wolbachia* in recent *D. paulistorum* samples from Southern Brazil. POA1 and POA10 are isofemale lines generated from collections in April 2003 in Porto Alegre City, Rio Grande do Sul State, kindly provided from Yong-Kyu Kim, Emory University, Atlanta, GA, USA. *Wolbachia*-specific *wsp* PCRs were performed on DNA of adults (a), 0–24 hrs embryos (e), and dissected ovaries (o). Similar to OR control flies (+), POA1 imagos harbor high-titer *Wolbachia*. Whereas standard *wsp* PCR detection systems are not sufficient to detect the symbiont in POA10 adults, they are clearly traceable in embryos (e) and ovaries (o). Intermediate *wsp*-signal intensity was obtained from hybrids of both sexes (AxO), derived from matings between low-titer AM females and high-titer OR males. The negative control was a *Wolbachia*-uninfected adult of the *D. simulans* strain STC.
(0.42 MB TIF)

Table S1 Variable nucleotide (A) and amino acid (B) sites in the *wsp* sequence of the closely related wAu-like *Wolbachia* strains of *Drosophila* and the Cherry fruit fly *Rhagoletis cerasi* (wCer2). *^a^* Position number 1 of the consensus sequence corresponds to position number 164 in the *wsp* sequence of *w*Au of *D. simulans* (AF020067). *^b^* Consensus *wsp* sequence obtained from the following *Wolbachia* strains: *w*Au of *D. simulans* Coffs Harbour [56]; *w*Wil of *D. willistoni*; *w*Pro SG1 & 2 of *D. prosaltans*; *w*Spt PLR1, 2 & BLI1 of *D. septentriosaltans* [59]; *w*Pau of *D. paulistorum* CA, TR and OR semispecies (yellow, this study), *w*Cer2 of *Rhagoletis cerasi* [109]; and *w*Mel of *D. melanogaster* [56]. *^c^* Designation of hypervariable regions (HVRs) and conserved regions (CR) of the WSP protein after [55].
(0.14 MB DOC)

Table S2 Mating choice assays performed on *D. paulistorum* semispecies before and after *Wolbachia* depletion via rifampicin. Mating choice assays performed on Amazonian (AM), Centroamerican (CA), Orinocan (OR) and the Andean Brazilian (AB; POA1 & POA10)) *D. paulistorum* semispecies before and after *Wolbachia* depletion via rifampicin.
(0.27 MB DOC)
