# Supplementary figures and images for: Infectious Speciation Revisited: Impact of Symbiont-Depletion on Female Fitness and Mating Behavior of Drosophila paulistorum
Source: PLoS Pathog. 2010 Dec 2;6(12):e1001214. doi: 10.1371/journal.ppat.1001214 (PMC2996333; doi:10.1371/journal.ppat.1001214)

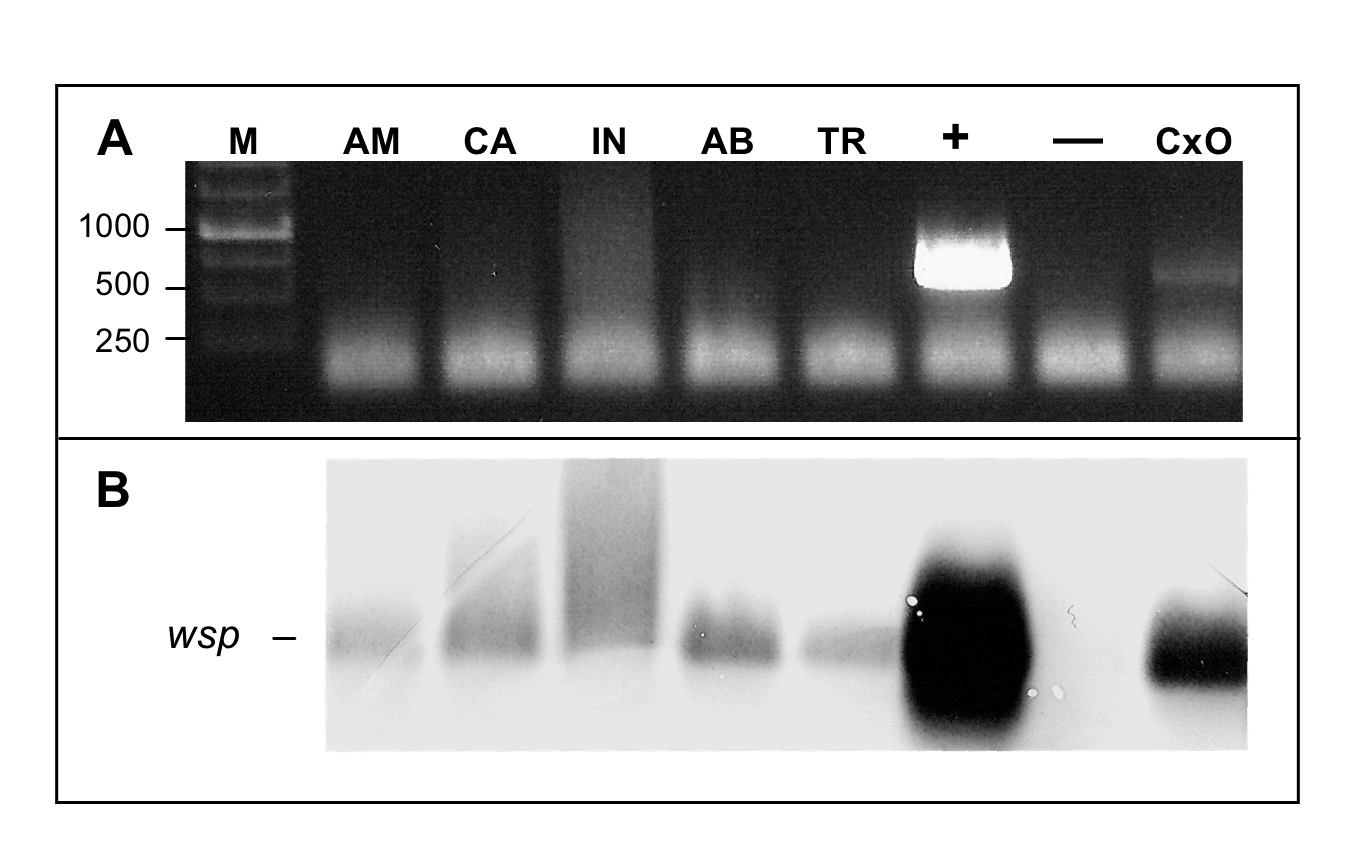

Supplement: Figure S1 — Wolbachia infection in adults of Drosophila paulistorum semispecies. Amazonian (AM); Centroamerican (CA); Interior (IN); Andean-Brazilian (AB); Transitional (TR); Orinocan (OR) semispecies and one F1 hybrid derived from crossings of CA females to OR males (CxO). (A) wsp-long PCR and (B) blot followed by hybridization with DIG-labeled wsp-probe (see Materials and Methods). (0.53 MB TIF) [file ppat.1001214.s001.tif]

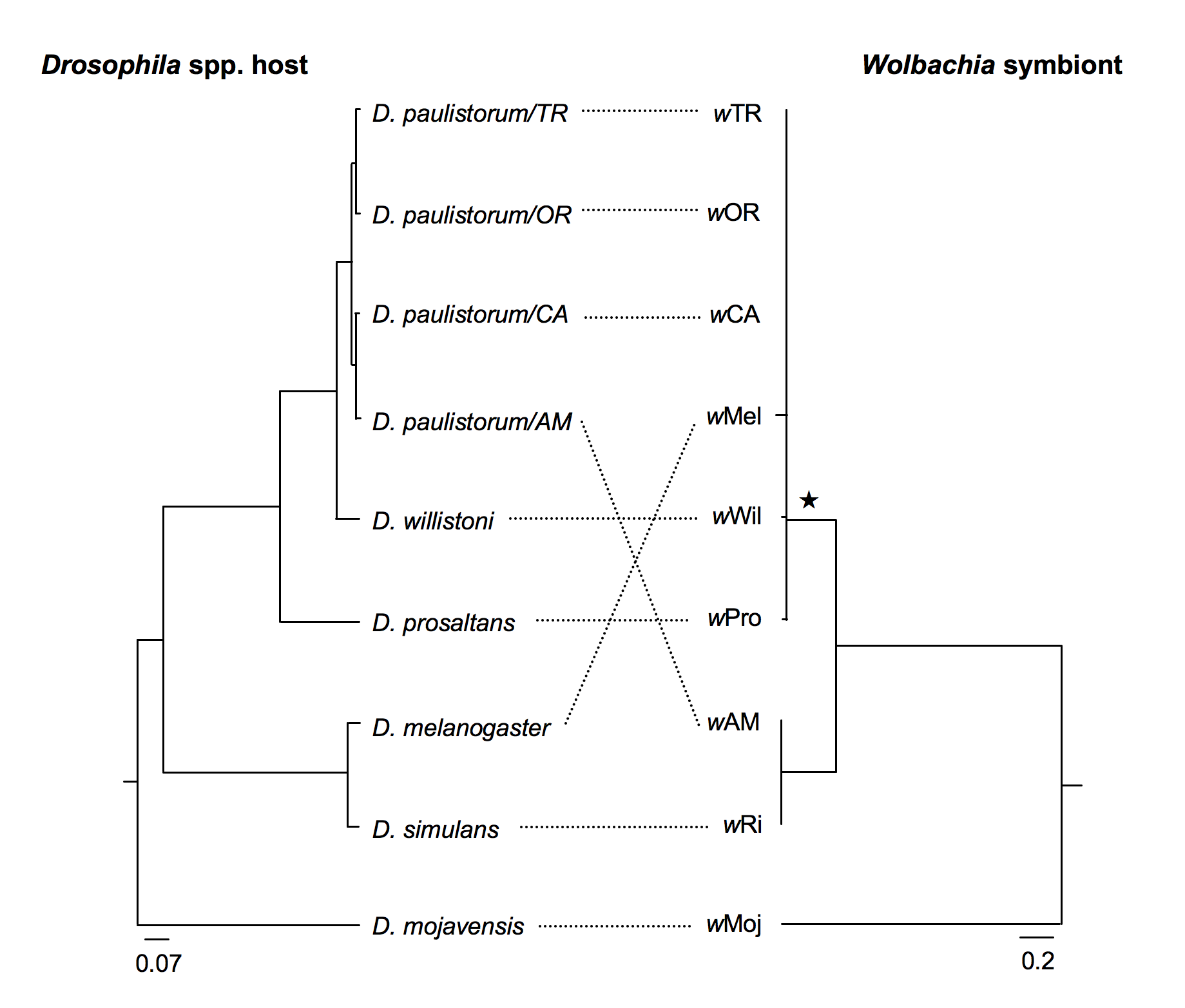

Supplement: Figure S2 — Co-cladogenesis of Wolbachia and host. Host tree (left) was calculated from nine Adh sequences of Drosophila species (AY364506; X57362; AE014134.5; AF045118; DWU95252; EU532128; EU532123; EU532127; EU532121) with 405 sites including gaps; method = average linkage (UPGMA). Wolbachia tree (right) was generated from nine wsp sequences (AY897491; AF020070; GQ924887; AY620227; AF620218; AF020063; GQ924889-90) with 652 sites including gaps; same method. Alignments (L-INS-i strategy) and tree calculation were performed using MAFFT 6.0 (http://mafft.cbrc.jp/alignment/server/); trees were edited using FigTreev1.2 (http://tree.bio.ed.ac.uk/software/figtree/). Asterisk indicates a region of rough resolution; for high resolution see SNP analysis in Table S1. (0.19 MB TIF) [file ppat.1001214.s002.tif]

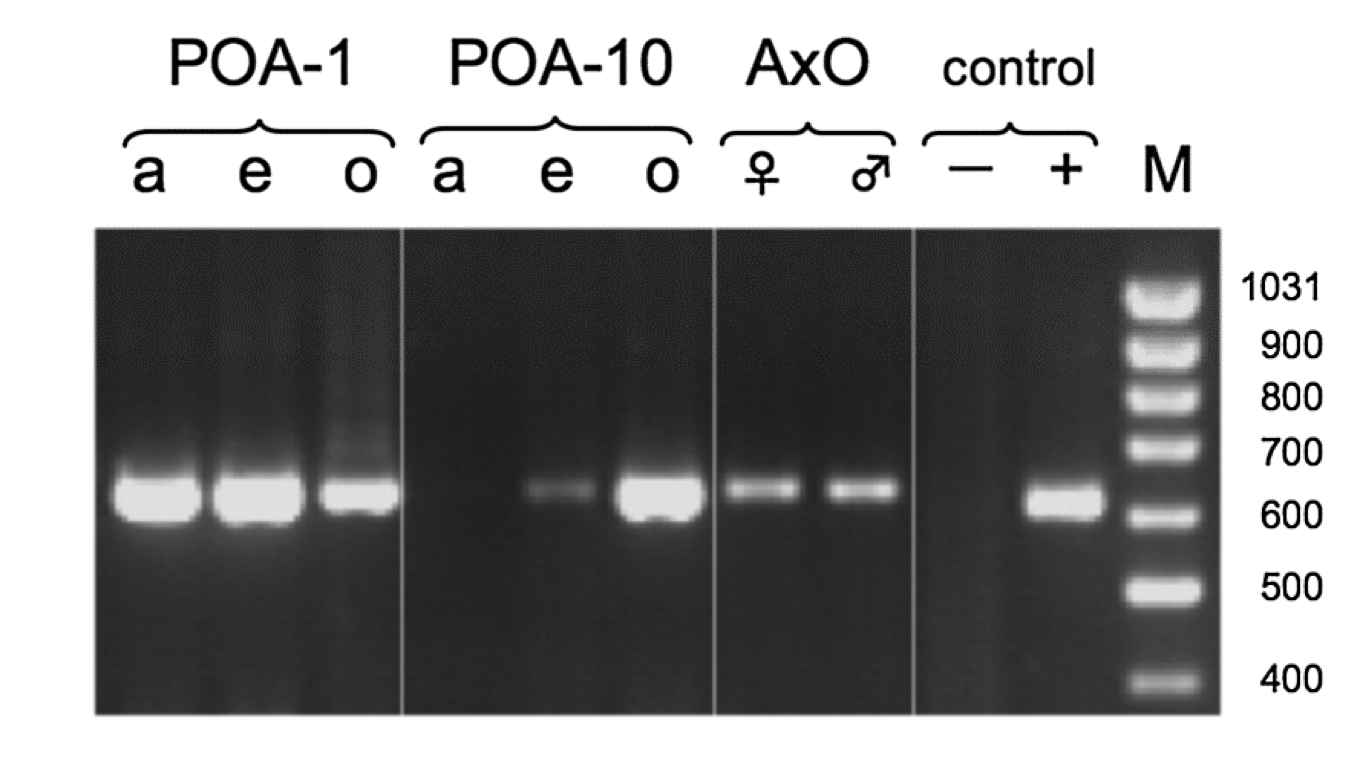

Supplement: Figure S3 — Natural presence of high- and low-titer Wolbachia in recent D. paulistorum samples from Southern Brazil. POA1 and POA10 are isofemale lines generated from collections in April 2003 in Porto Alegre City, Rio Grande do Sul State, kindly provided from Yong-Kyu Kim, Emory University, Atlanta, GA, USA. Wolbachia-specific wsp PCRs were performed on DNA of adults (a), 0–24 hrs embryos (e), and dissected ovaries (o). Similar to OR control flies (+), POA1 imagos harbor high-titer Wolbachia. Whereas standard wsp PCR detection systems are not sufficient to detect the symbiont in POA10 adults, they are clearly traceable in embryos (e) and ovaries (o). Intermediate wsp-signal intensity was obtained from hybrids of both sexes (AxO), derived from matings between low-titer AM females and high-titer OR males. The negative control was a Wolbachia-uninfected adult of the D. simulans strain STC. (0.42 MB TIF) [file ppat.1001214.s003.tif]
