# Supplementary material for: Infectious Speciation Revisited: Impact of Symbiont-Depletion on Female Fitness and Mating Behavior of Drosophila paulistorum
Source: PLoS Pathog. 2010 Dec 2;6(12):e1001214. doi: 10.1371/journal.ppat.1001214 (PMC2996333; doi:10.1371/journal.ppat.1001214)
Supplement: Table S1 — Variable nucleotide (A) and amino acid (B) sites in the wsp sequence of the closely related wAu-like Wolbachia strains of Drosophila and the Cherry fruit fly Rhagoletis cerasi (wCer2). a Position number 1 of the consensus sequence corresponds to position number 164 in the wsp sequence of wAu of D. simulans (AF020067). b Consensus wsp sequence obtained from the following Wolbachia strains: wAu of D. simulans Coffs Harbour [56]; wWil of D. willistoni; wPro SG1 & 2 of D. prosaltans; wSpt PLR1, 2 & BLI1 of D. septentriosaltans [59]; wPau of D. paulistorum CA, TR and OR semispecies (yellow, this study), wCer2 of Rhagoletis cerasi [109]; and wMel of D. melanogaster [56]. c Designation of hypervariable regions (HVRs) and conserved regions (CR) of the WSP protein after [55]. (0.14 MB DOC) [file ppat.1001214.s004.doc]

| **A** |  |  |  |  |  |  |  |  |  |  |  |  |  |  |  |
| --- | --- | --- | --- | --- | --- | --- | --- | --- | --- | --- | --- | --- | --- | --- | --- |
|  |  |  |  |  |  |  |  |  |  |  |  |  |  |  |  |
| variable positions in *wsp* **DNA***^a^* | 68 | 70 | 104 | 170 | 211 | 263 | 301 | 304 | 340 | 377 | 472 | 520 | 529 | 536 | 538 |
| consensus*^b^* | **C** | **T** | **A** | **A** | **G** | **G** | **A** | **C** | **G** | **A** | **T** | **G** | **A** | **T** | **A** |
| *w*Au | **.** | **.** | **.** | **.** | **.** | **.** | **.** | **.** | **A** | **.** | **.** | **.** | **.** | **.** | **.** |
| *w*Wil | **.** | **.** | **.** | **.** | **.** | **.** | **.** | **.** | **A** | **.** | **.** | **.** | **.** | **.** | **.** |
| *w*Pro SG1 | **G** | **C** | **.** | **.** | **.** | **A** | **.** | **.** | **.** | **.** | **.** | **.** | **.** | **.** | **.** |
| *w*Pro SG2 | **G** | **C** | **.** | **.** | **.** | **.** | **.** | **.** | **.** | **.** | **.** | **.** | **.** | **.** | **.** |
| *w*Spt PLR1 | **.** | **C** | **.** | **.** | **.** | **.** | **.** | **.** | **.** | **G** | **.** | **.** | **.** | **.** | **.** |
| *w*Spt PLR2 | **.** | **C** | **.** | **.** | **.** | **.** | **.** | **.** | **.** | **.** | **.** | **.** | **.** | **.** | **.** |
| *w*Spt BCI1 | **.** | **.** | **.** | **.** | **.** | **.** | **.** | **.** | **.** | **.** | **.** | **.** | **.** | **.** | **.** |
| *w*Pau-CA | **.** | **.** | **G** | **.** | **.** | **.** | **.** | **.** | **.** | **.** | **.** | **.** | **.** | **.** | **.** |
| *w*Pau-TR | **.** |  | **G** | **.** | **.** | **.** | **.** | **.** | **.** | **.** | **.** | **.** | **.** | **.** | **.** |
| *w*Pau-OR | **.** | **.** | **.** | **.** | **.** | **.** | **.** | **.** | **.** | **.** | **.** | **.** | **.** | **.** | **.** |
| *w*Cer2 | **.** | **.** | **.** | **.** | **.** | **.** | **.** | **.** | **.** | **.** | **.** | **.** | **.** | **.** | **.** |
| *w*Mel | **.** | **.** | **.** | **.** | **.** | **.** | **.** | **.** | **.** | **.** | **.** | **A** | **G** | **C** | **G** |
|  |  |  |  |  |  |  |  |  |  |  |  |  |  |  |  |
| **B** |  |  |  |  |  |  |  |  |  |  |  |  |  |  |  |
|  |  |  |  |  |  |  |  |  |  |  |  |  |  |  |  |
| WSP domain*^c^* |  | HVR1 | | CR2 | HVR  2 | CR3 |  |  |  | HVR  3 | CR4 |  | HVR4 | |  |
| variable positions in WSP **protein** | 23 | 24 | 35 | 57 | 71 | 88 | 101 | 102 | 114 | 126 | 158 | 174 | 177 | 179 | 180 |
| consensus*^b^* | **T** | **Y** | **K** | **G** | **V** | **G** | **M** | **P** | **A** | **D** | **F** | **D** | **R** | **V** | **T** |
| *w*Au | **.** | **.** | **.** | **.** | **.** | **.** | **.** | **.** | **T** | **.** | **.** | **.** | **.** | **.** | **.** |
| *w*Wil | **.** | **.** | **.** | **.** | **.** | **.** | **.** | **.** | **T** | **.** | **.** | **.** | **.** | **.** | **.** |
| *w*Pro SG1 | **S** | **H** | **.** | **.** | **.** | **E** | **.** | **.** | **.** | **.** | **.** | **.** | **.** | **.** | **.** |
| *w*Pro SG2 | **S** | **H** | **.** | **.** | **.** | **.** | **.** | **.** | **.** | **.** | **.** | **.** | **.** | **.** | **.** |
| *w*Spt PLR1 | **.** | **H** | **.** | **.** | **.** | **.** | **.** | **.** | **.** | **G** | **.** | **.** | **.** | **.** | **.** |
| *w*Spt PLR2 | **.** | **H** | **.** | **.** | **.** | **.** | **.** | **.** | **.** | **.** | **.** | **.** | **.** | **.** | **.** |
| *w*Spt BCI1 | **.** | **.** | **.** | **.** | **.** | **.** | **.** | **.** | **.** | **.** | **.** | **.** | **.** | **.** | **.** |
| *w*Pau-CA | **.** | **.** | **R** | **.** | **.** | **.** | **.** | **.** | **.** | **.** | **.** | **.** | **.** | **.** | **.** |
| *w*Pau-TR | **.** | **.** | **R** | **.** | **.** | **.** | **.** | **.** | **.** | **.** | **.** | **.** | **.** | **.** | **.** |
| *w*Pau-OR | **.** | **.** | **.** | **.** | **.** | **.** | **.** | **.** | **.** | **.** | **.** | **.** | **.** | **.** | **.** |
| *w*Cer2 | **.** | **.** | **.** | **.** | **.** | **.** | **.** | **.** | **.** | **.** | **.** | **.** | **.** | **.** | **.** |
| *w*Mel | **.** | **.** | **.** | **.** | **.** | **.** | **.** | **.** | **.** | **.** | **.** | **N** | **G** | **A** | **A** |
