# Supplementary material for: Infectious Speciation Revisited: Impact of Symbiont-Depletion on Female Fitness and Mating Behavior of Drosophila paulistorum
Source: PLoS Pathog. 2010 Dec 2;6(12):e1001214. doi: 10.1371/journal.ppat.1001214 (PMC2996333; doi:10.1371/journal.ppat.1001214)
Supplement: Table S2 — Mating choice assays performed on D. paulistorum semispecies before and after Wolbachia depletion via rifampicin. Mating choice assays performed on Amazonian (AM), Centroamerican (CA), Orinocan (OR) and the Andean Brazilian (AB; POA1 & POA10)) D. paulistorum semispecies before and after Wolbachia depletion via rifampicin. (0.27 MB DOC) [file ppat.1001214.s005.doc]

| **Nr. 1: AM^U^*;* OR^U^ (both untreated)** | | | | | | | |
| --- | --- | --- | --- | --- | --- | --- | --- |
|  | **^2^**1a | 1b | 2a | 2b | 3a | N | Freq. |
| AM^U^ x AM^U^ | 9 (0.750) | 8 (0.667) | 10 (0.834) | 11 (0.917) | 11 (0.917) | 49 | 0.41 |
| AM^U^ x OR^U^ | 3 (0.250) | 4 (0.333) | 2 (0.167) | 1 (0.083) | 1 (0.083) | 11 | 0.09 |
| OR^U^ x AM^U^ | 0 (0.000) | 2 (0.167) | 0 (0.000) | 2 (0.167) | 3 (0.250) | 7 | 0.06 |
| OR^U^ x OR^U^ | 12 (1.000) | 10 (0.834) | 12 (1.000) | 10 (0.834) | 9 (0.750) | 53 | 0.44 |
|  | 24 | 24 | 24 | 24 | 24 | 120 |  |
| **^3^** SII (S.E.) = 0.70 (0.0043); **^4^** SII (S.E.) = 0.70 (0.08) | | | | | | | |
|  | | | | | | | |
| **Nr. 2: AM^T^*;* OR^T^ (both treated 0.01% Rif)** | | | | | | | |
|  | 1a | 1b | 2a | 2b | 3a | N | Freq. |
| AM^T^ x AM^T^ | 10 (0.834) | 11 (0.917) | 10 (0.834) | 12 (1.000) | 11 (0.917) | 54 | 0.45 |
| AM^T^ x OR^T^ | 2 (0.167) | 1 (0.083) | 2 (0.167) | 0 (0.000) | 1 (0.083) | 6 | 0.05 |
| OR^T^ x AM^T^ | 0 (0.000) | 0 (0.000) | 1 (0.083) | 0 (0.000) | 1 (0.083) | 2 | 0.02 |
| OR^T^ x OR^T^ | 12 (1.000) | 12 (1.000) | 11 (0.917) | 12 (1.000) | 11 (0.917) | 58 | 0.48 |
|  | 24 | 24 | 24 | 24 | 24 | 120 |  |
| SII (S.E.) = 0.86 (0.002); SII (S.E.) = 0.87 (0.02); *P* = 0.0599 N.S. | | | | | | | |
|  | | | | | | | |
| **Nr. 3: AM^T^*;* OR^T^ (both treated 0.1% Rif)** | | | | | | | |
|  | 1a | 1b | 2a | 2b | a | N | Freq. |
| AM^T^ x AM^T^ | 12 (1.000) | 10 (0.834) | 12 (1.000) | 11 (0.917) | 10 (0.834) | 55 | 0.46 |
| AM^T^ x OR^T^ | 0 (0.000) | 2 (0.167) | 0 (0.000) | 1 (0.083) | 2 (0.167) | 5 | 0.04 |
| OR^T^ x AM^T^ | 0 (0.000) | 0 (0.000) | 1 (0.083) | 2 (0.167) | 2 (0.167) | 5 | 0.04 |
| OR^T^ x OR^T^ | 12 (1.000) | 12 (1.000) | 11 (0.917) | 10 (0.834) | 10 (0.834) | 55 | 0.46 |
|  | 24 | 24 | 24 | 24 | 24 | 120 |  |
| SII (S.E.) = 0.84 (0.0025); SII (S.E.) = 0.83 (0.05); *P* = 0.1584 N.S. | | | | | | | |
|  | | | | | | | |
| **Nr. 4: AM^T^; OR^T^ (both treated 0.2% Rif)** | | | | | | | |
|  | 1a | 1b | 2a | 2b | 3a | N | Freq. |
| AM^T^ x AM^T^ | 5 (0.417) | 4 (0.333) | 3 (0.250) | 5 (0.417) | 9 (0.750) | 26 | 0.22 |
| AM^T^ x OR^T^ | 7 (0.583) | 8 (0.667) | 9 (0.750) | 7 (0.583) | 3 (0.250) | 34 | 0.28 |
| OR^T^ x AM^T^ | 4 (0.333) | 5 (0.417) | 2 (0.167) | 6 (0.500) | 1 (0.083) | 18 | 0.15 |
| OR^T^ x OR^T^ | 8 (0.667) | 7 (0.583) | 10 (0.834) | 6 (0.500) | 11 (0.917) | 42 | 0.35 |
|  | 24 | 24 | 24 | 24 | 24 | 120 |  |
| SII (S.E.) = 0.13 (0.008); *P* < 0.0001 *** | | | | | | | |
|  | | | | | | | |
| **Nr. 5: OR^T^ (0.01% Rif); AM^U^ (untreated)** | | | | | | | |
|  | 1a | 1b | 2a | 2b | 3a | N | Freq. |
| AM^U^ x AM^U^ | 8 (0.667) | 10 (0.834) | 10 (0.834) | 11 (0.917) | 10 (0.834) | 49 | 0.41 |
| AM^U^ x OR^T^ | 4 (0.333) | 2 (0.167) | 2 (0.167) | 1 (0.083) | 2 (0.167) | 11 | 0.09 |
| OR^T^ x AM^U^ | 2 (0.167) | 4 (0.333) | 4 (0.333) | 4 (0.333) | 3 (0.250) | 17 | 0.14 |
| OR^T^ x OR^T^ | 10 (0.834) | 8 (0.667) | 8 (0.667) | 8 (0.667) | 9 (0.750) | 43 | 0.36 |
| SII (S.E.) = 0.54 (0.005); *P* = 0.8565 N.S. | | | | | | | |

| **Nr. 6: OR^T^ (0.1% Rif); AM^U^ (untreated)** | | | | | | | |
| --- | --- | --- | --- | --- | --- | --- | --- |
|  | 1a | 1b | 2a | 2b | 3a | N | Freq. |
| AM^U^ x AM^U^ | 12 (1.000) | 9 (0.750) | 11 (0.917) | 12 (1.000) | 6 (0.500) | 50 | 0.42 |
| AM^U^ x OR^T^ | 0 (0.000) | 3 (0.250) | 1 (0.083) | 0 (0.000) | 6 (0.500) | 10 | 0.08 |
| OR^T^ x AM^U^ | 6 (0.500) | 7 (0.583) | 3 (0.250) | 3 (0.250) | 2 (0.167) | 21 | 0.18 |
| OR^T^ x OR^T^ | 6 (0.500) | 5 (0.417) | 9 (0.750) | 9 (0.750) | 10 (0.834) | 39 | 0.32 |
|  | 24 | 24 | 24 | 24 | 24 | 120 |  |
| SII (S.E.) = 0.48 (0.007); SII (SE) = 0.48 (0.08); *P* = 0.0539 * | | | | | | | |
|  | | | | | | | |
| **Nr. 7: OR^T^ (0.2% Rif); AM^U^ (untreated)** | | | | | | | |
|  | 1a | 1b | 2a | 2b | 3a | N | Freq. |
| AM^U^ x AM^U^ | 8 (0.667) | 9 (0.750) | 9 (0.750) | 9 (0.750) | 9 (0.750) | 44 | 0.37 |
| AM^U^ x OR^T^ | 4 (0.333) | 3 (0.250) | 3 (0.250) | 3 (0.250) | 3 (0.250) | 16 | 0.13 |
| OR^T^ x AM^U^ | 5 (0.417) | 5 (0.417) | 4 (0.333) | 5 (0.417) | 5 (0.417) | 24 | 0.20 |
| OR^T^ x OR^T^ | 7 (0.583) | 7 (0.583) | 8 (0.667) | 7 (0.583) | 7 (0.583) | 36 | 0.30 |
|  | 24 | 24 | 24 | 24 | 24 | 120 |  |
| SII (S.E.) = 0.33 (0.008); *P* = 0.0014 ** | | | | | | | |
|  | | | | | | | |
| **Nr. 8: AM^T^ (0.01% Rif); OR^U^ (untreated)** | | | | | | | |
|  | 1a | 1b | 2a | 2b | 3a | N | Freq. |
| AM^T^ x AM^T^ | 3 (0.250) | 1 (0.083) | 4 (0.333) | 7 (0.583) | 5 (0.417) | 20 | 0.17 |
| AM^T^ x OR^U^ | 9 (0.750) | 11 (0.917) | 8 (0.667) | 5 (0.417) | 7 (0.583) | 40 | 0.33 |
| OR^U^ x AM^T^ | 2 (0.167) | 2 (0.167) | 7 (0.583) | 6 (0.500) | 3 (0.250) | 17 | 0.14 |
| OR^U^ x OR^U^ | 10 (0.834) | 10 (0.834) | 8 (0.667) | 6 (0.500) | 9 (0.750) | 43 | 0.36 |
|  | 24 | 24 | 24 | 24 | 24 | 120 |  |
| SII (S.E.) = 0.06 (0.0083); SII (SE) = 0.05 (0.09); *P* < 0.0001 *** | | | | | | | |
|  | | | | | | | |
| **Nr. 9: AM^T^ (0.1% Rif); OR^U^ (untreated)** | | | | | | | |
|  | 1a | 1b | 2a | 2b | 3a | N | Freq. |
| AM^T^ x AM^T^ | 5 (0.417) | 5 (0.417) | 8 (0.667) | 7 (0.583) | 7 (0.583) | 32 | 0.27 |
| AM^T^ x OR^U^ | 7 (0.583) | 7 (0.583) | 4 (0.333) | 5 (0.417) | 5 (0.417) | 28 | 0.23 |
| OR^U^ x AM^T^ | 6 (0.500) | 5 (0.417) | 6 (0.500) | 2 (0.167) | 3 (0.250) | 22 | 0.18 |
| OR^U^ x OR^U^ | 6 (0.500) | 7 (0.583) | 6 (0.500) | 10 (0.834) | 9 (0.750) | 38 | 0.32 |
|  | 24 | 24 | 24 | 24 | 24 | 120 |  |
| SII (S.E.) = 0.18 (0.0081); SII (SE) = 0.17 (0.09); *P* < 0.0001 *** | | | | | | | |
|  | | | | | | | |
| **Nr. 10: AM^U^; CA^U^ both untreated** | | | | | | | |
|  | 1a | 1b | 2a | 2b | 3a | N | Freq. |
| AM^U^ x AM^U^ | 11 (0.917) | 11 (0.917) | 11 (0.917) | 12 (1.000) | 11 (0.917) | 56 | 0.47 |
| AM^U^ x CA^U^ | 1 (0.083) | 1 (0.083) | 1 (0.083) | 0 (0.000) | 1 (0.083) | 4 | 0.03 |
| CA^U^ x AM^U^ | 0 (0.000) | 0 (0.000) | 2 (0.167) | 0 (0.000) | 0 (0.000) | 2 | 0.02 |
| CA^U^ x CA^U^ | 12 (1.000) | 12 (1.000) | 10 (0.834) | 12 (1.000) | 12 (1.000) | 58 | 0.48 |
| SII (S.E.) = 0.90 (0.0016); SII (SE) = 0.90 (0.04) | | | | | | | |

| **Nr. 11: AM^T^; CA^T^ (both treated 0.1% Rif)** | | | | | | | | | | | | | | |
| --- | --- | --- | --- | --- | --- | --- | --- | --- | --- | --- | --- | --- | --- | --- |
|  | 1a | | 1b | | 2a | | 2b | | 3a | | N | | Freq. | |
| AM^T^ x AM^T^ | 9 (0.750) | | 8 (0.667) | | 9 (0.750) | | 10 (0.834) | | 10 (0.834) | | 46 | | 0.38 | |
| AM^T^ x CA^T^ | 3 (0.250) | | 4 (0.333) | | 3 (0.250) | | 2 (0.167) | | 2 (0.167) | | 14 | | 0.12 | |
| CA^T^ x AM^T^ | 6 (0.500) | | 4 (0.333) | | 5 (0.417) | | 5 (0.417) | | 3 (0.250) | | 23 | | 0.19 | |
| CA^T^ x CA^T^ | 6 (0.500) | | 8 (0.667) | | 7 (0.583) | | 7 (0.583) | | 9 (0.750) | | 37 | | 0.31 | |
|  | 24 | | 24 | | 24 | | 24 | | 24 | | 120 | |  | |
| SII (S.E.) = 0.38 (0.0071); SII (SE) = 0.38 (0.08); *P* < 0.0001 *** | | | | | | | | | | | | | | |
|  | | | | | | | | | | | | | | |
| **Nr. 12: AM^T^ (0.1% Rif); CA^U^ (untreated)** | | | | | | | | | | | | | | |
|  | 1a | | 1b | | 2a | | 2b | | 3a | | N | | Freq. | |
| AM^T^ x AM^T^ | 9 (0.750) | | 10 (0.834) | | 10 (0.834) | | 8 (0.667) | | 7 (0.583) | | 44 | | 0.37 | |
| AM^T^ x CA^U^ | 3 (0.250) | | 2 (0.167) | | 2 (0.167) | | 4 (0.333) | | 5 (0.417) | | 16 | | 0.13 | |
| CA^U^ x AM^T^ | 3 (0.250) | | 3 (0.250) | | 4 (0.333) | | 4 (0.333) | | 3 (0.250) | | 17 | | 0.14 | |
| CA^U^ x CA^U^ | 9 (0.750) | | 9 (0.750) | | 8 (0.667) | | 8 (0.667) | | 9 (0.750) | | 43 | | 0.36 | |
|  | 24 | | 24 | | 24 | | 24 | | 24 | | 120 | | 24 | |
| SII (S.E.) = 0.46 (0.0066); SII (SE) = 0.45 (0.08); *P* < 0.0001 *** | | | | | | | | | | | | | | |
|  | | | | | | | | | | | | | | |
| **Nr. 13: CA^T^ (0.1% Rif); AM^U^ (untreated)** | | | | | | | | | | | | | | |
|  | 1a | | 1b | | 2a | | 2b | | 3a | | N | | Freq. | |
| AM^U^ x AM^U^ | 8 (0.667) | | 9 (0.750) | | 10 (0.834) | | 11 (0.917) | | 8 (0.667) | | 46 | | 0.38 | |
| AM^U^ x CA^T^ | 4 (0.333) | | 3 (0.250) | | 2 (0.167) | | 1 (0.083) | | 4 (0.333) | | 14 | | 0.12 | |
| CA^T^ x AM^U^ | 3 (0.250) | | 5 (0.417) | | 3 (0.250) | | 5 (0.417) | | 3 (0.250) | | 19 | | 0.16 | |
| CA^T^ x CA^T^ | 9 (0.750) | | 7 (0.583) | | 9 (0.750) | | 7 (0.583) | | 9 (0.750) | | 41 | | 0.34 | |
|  | 24 | | 24 | | 24 | | 24 | | 24 | | 120 | |  | |
| SII (S.E.) = 0.44 (0.0067); SII (SE) = 0.45 (0.08); *P* < 0.0001 *** | | | | | | | | | | | | | | |
|  | | | | | | | | | | | | | | |
| **Nr. 14: POA1^U^; CA^U^ both untreated** | | | | | | | | | | | | | | |
|  | | 1a | | 1b | | 2a | | 2b | | 3a | | N | | Freq. |
| CA^U^ x CA^U^ | | 12 (1.000) | | 11 (0.917) | | 11 (0.917) | | 12 (1.000) | | 12 (1.000) | | 58 | | 0.48 |
| CA^U^ x POA1^U^ | | 0 (0.000) | | 1 (0.083) | | 1 (0.083) | | 0 (0.000) | | 0 (0.000) | | 2 | | 0.02 |
| POA1^U^ x CA^U^ | | 0 (0.000) | | 1 (0.083) | | 0 (0.000) | | 1 (0.083) | | 0 (0.000) | | 2 | | 0.02 |
| POA1^U^ x POA1^U^ | | 12 (1.000) | | 11 (0.917) | | 12 (1.000) | | 11 (0.917) | | 12 (1.000) | | 58 | | 0.48 |
|  | | 24 | | 24 | | 24 | | 24 | | 24 | | 120 | |  |
| SII (S.E.) = 0.92 (0.001) | | | | | | | | | | | | | | |
|  | | | | | | | | | | | | | | |
| **Nr. 15: POA1^T^ (0.2% Rif); CA^U^ (untreated)** | | | | | | | | | | | | | | |
|  | | 1a | | 1b | | 2a | | 2b | | 3a | | N | | Freq. |
| CA^U^ x CA^U^ | | 8 (0.667) | | 9 (0.750) | | 5 (0.417) | | 12 (1.000) | | 10 (0.834) | | 44 | | 0.37 |
| CA^U^ x POA1^T^ | | 4 (0.333) | | 3 (0.250) | | 7 (0.583) | | 0 (0.000) | | 2 (0.167) | | 16 | | 0.13 |
| POA1^T^ x CA^U^ | | 5 (0.417) | | 0 (0.000) | | 4 (0.333) | | 3 (0.250) | | 3 (0.250) | | 15 | | 0.13 |
| POA1^T^ x POA1^T^ | | 7 (0.583) | | 12 (1.000) | | 8 (0.667) | | 9 (0.750) | | 9 (0.750) | | 45 | | 0.38 |
| SII (S.E.) = 0.49 (0.006); *P* < 0.0001 *** | | | | | | | | | | | | | | |

| **Nr. 16: POA10^U^; CA^U^ (both untreated)** | | | | | | | |
| --- | --- | --- | --- | --- | --- | --- | --- |
|  | 1a | 1b | 2a | 2b | 3a | N | Freq. |
| CA^U^ x CA^U^ | 12 (1.000) | 12 (1.000) | 10 (0.834) | 12 (1.000) | 11 (0.917) | 57 | 0.48 |
| CA^U^ x POA10^U^ | 0 (0.000) | 0 (0.000) | 2 (0.167) | 0 (0.000) | 1 (0.083) | 3 | 0.03 |
| POA10^U^ x CA^U^ | 0 (0.000) | 0 (0.000) | 0 (0.000) | 0 (0.000) | 0 (0.000) | 0 | 0.00 |
| POA10^U^ x POA10^U^ | 12 (1.000) | 12 (1.000) | 12 (1.000) | 12 (1.000) | 12 (1.000) | 60 | 0.50 |
| SII (S.E.) = 0.95 (0.001) | | | | | | | |
|  | | | | | | | |
| **Nr. 17: POA10^T^; (0.2% Rif); CA^U^ (untreated)** | | | | | | | |
|  | 1a | 1b | 2a | 2b | 3a | N | Freq. |
| CA^U^ x CA^U^ | 6 (0.500) | 10 (0.834) | 12 (1.000) | 7 (0.583) | 10 (0.834) | 45 | 0.38 |
| CA^U^ x POA10^T^ | 6 (0.500) | 2 (0.167) | 0 (0.000) | 5 (0.417) | 2 (0.167) | 15 | 0.13 |
| POA10^T^ x CA^U^ | 2 (0.167) | 6 (0.500) | 6 (0.500) | 4 (0.333) | 6 (0.500) | 24 | 0.20 |
| POA10^T^ x POA10^T^ | 10 (0.834) | 6 (0.500) | 6 (0.500) | 8 (0.667) | 6 (0.500) | 36 | 0.30 |
|  | 24 | 24 | 24 | 24 | 24 | 120 |  |
| SII (S.E.) = 0.35 (0.007); *P* < 0.0001 *** | | | | | | | |
|  | | | | | | | |
| **Nr. 18: *D. sim*^T^ (0.2% Rif); *D. sim*^U^ (untreated)** | | | | | | | |
|  | 1a | 1b | 2a | 2b | 3a | N | Freq. |
| *D. sim*^U^ x *D. sim*^U^ | 7 (0.583) | 6 (0.500) | 7 (0.583) | 4 (0.333) | 7 (0.583) | 31 | 0.26 |
| *D. sim*^U^ x *D. sim*^T^ | 5 (0.417) | 6 (0.500) | 5 (0.417) | 8 (0.667) | 5 (0.417) | 29 | 0.24 |
| *D. sim*^T^ x *D. sim*^U^ | 6 (0.500) | 4 (0.333) | 3 (0.250) | 8 (0.667) | 8 (0.667) | 29 | 0.24 |
| *D. sim*^T^ x *D. sim*^T^ | 6 (0.500) | 8 (0.667) | 9 (0.750) | 4 (0.333) | 4 (0.333) | 31 | 0.26 |
|  | 24 | 24 | 24 | 24 | 24 | 120 |  |
| SII (S.E.) = 0.04 (0.01); *P* = 0.1835 N.S. | | | | | | | |

**^1^** Pairs of combinations (females first) between Amazonian (AM), Centroamerican (CA), Orinocan (OR) semispecies, and Andean Brazilian (AB), lines POA1and POA10. **^U^** = untreated; **^T^** = treated with antibiotics.

**^2^** For each array five replicas (1a - 3a) and 120 matings were scored (12A ♀♀ + 12B ♀♀ + 12A ♂♂ + 12B ♂♂ differentiated by rotated wing clips) for each row, totaling 2160 matings. Absolute numbers of successful matings are given first, followed in parenthesis by mating frequency out of 12 females.

^N^ number of successful matings for each combination out of 120 females per assay

**^3^** Sexual Isolation Index (SII) and standard error (S.E.) were determined following [**72**]; and

**^4^** joint isolation index after [**107**]
